# Non-coding RNA 7SK drives tumor resistance by coupling local oncogenic activation with global transcriptional repression

**DOI:** 10.1371/journal.pcbi.1014749

**Published:** 2026-09-08

**Authors:** Ping Xu, Zile Luo, Xi Chen, Zhongyang Yuan, Le Cheng, Yi Yang, Hongcheng Lin, Qiong Zhang, Pengfei Qin

**Affiliations:** 1 BGI Research, Chongqing, China; 2 BGI College & Henan Institute of Medical and Pharmaceutical Sciences, Zhengzhou University, Zhengzhou, China; 3 College of Life Sciences, University of Chinese Academy of Sciences, Beijing, China; 4 State Key Laboratory of Genome and Multi-omics Technologies, BGI Research, Shenzhen, China; 5 BGI Research, Kunming, China; 6 Department of General Surgery (Department of Coloproctology), The Sixth Affiliated Hospital, Sun Yat-sen University, Guangzhou, China; 7 Guangdong Provincial Key Laboratory of Colorectal and Pelvic Floor Diseases, The Sixth Affiliated Hospital, Sun Yat-sen University, Guangzhou, China; 8 Biomedical Innovation Center, The Sixth Affiliated Hospital, Sun Yat-sen University, Guangzhou, China; 9 Department of Obstetrics and Reproductive Medicine, Affiliated Hospital of Yunnan University, Kunming, Yunnan, China; 10 Tianjian Laboratory of Advanced Biomedical Sciences, Institute of Advanced Biomedical Sciences, Zhengzhou University, Zhengzhou, Henan, China; Jilin University, CHINA

## Abstract

The conserved non-coding RNA 7SK is a well-established global transcriptional repressor, yet its context-specific functions in cancer and therapy resistance remain paradoxical. Here, we resolve this paradox by uncovering a dual-axis mechanism through which 7SK drives colorectal cancer (CRC) resistance. By integrating single-cell multi-omics with functional assays, we demonstrate that 7SK not only selectively activates the JUN transcriptional network to fuel tumor proliferation but also reduces global transcriptional entropy to stabilize an immunosuppressive microenvironment and promote immune escape. This “local activation-global suppression” paradigm is conserved across multiple cancer types, positioning 7SK as a potential pan-cancer therapeutic target. Our findings reveal 7SK as a dynamic modulator that balances oncogene-specific transcription with global transcriptional suppression across cancers, providing a new framework for understanding and targeting ncRNA-mediated resistance.

## Author summary

Why does a molecule known for shutting down gene activity actually help tumors survive chemotherapy? This intriguing paradox motivated our investigation. Using single-cell technologies to analyze patient tumor samples, we found that in chemotherapy-resistant colorectal cancer cells, the non-coding RNA 7SK selectively amps up a specific set of proliferation-driving genes through the JUN network, pressing the accelerator on tumor growth. At the same time, it broadly dampens overall gene expression disorder, which quiets immune alarm signals and creates a “cold” tumor environment that evades immune attack. This dual strategy of local activation paired with global suppression helps resistant clones thrive under therapeutic pressure. Remarkably, we observed the same mechanism operating across multiple cancer types, suggesting that 7SK’s role is not a cancer-specific quirk but a conserved survival program. Our findings reframe 7SK from a simple repressor to a master conductor of transcriptional balance in cancer. By uncovering 7SK’s dual role, we lay a foundation for future therapeutic strategies aimed at disrupting this mechanism to overcome drug resistance.

## Introduction

Non-coding RNAs (ncRNAs) play pivotal roles in gene regulation and disease progression, yet their functions in tumor evolution and therapy resistance remain incompletely understood due to their inherent diversity and complex modes of action [1–3]. Among them, the highly conserved ncRNA 7SK has been classically characterized as a global transcriptional repressor. By sequestering the positive transcription elongation factor P-TEFb, 7SK potently inhibits RNA Polymerase II elongation, thereby dampening transcriptional output across the genome [4–10]. However, emerging evidence from cancer contexts presents a conceptual dilemma: despite this global repressive role, 7SK appears to be required for the expression of specific tumor-promoting genes and pathways [11–16]. This suggests a hitherto unresolved duality: the capacity for 7SK to integrate widespread transcriptional repression with selective local activation. The mechanistic basis and functional consequences of this putative duality in driving therapy resistance remain almost entirely elusive, obscured by ongoing debates over its oncogenic versus tumor-suppressive roles [11,14,17,18].

A central knowledge gap concerns the potential duality of 7SK function, its ability to integrate global transcriptional repression with selective local activation of tumor-promoting pathways [7,9,12,16,18,19]. On one hand, 7SK restricts transcriptional activity through P-TEFb sequestration, thereby reducing the diversity and disorder of gene expression, a phenomenon described as transcriptional entropy reduction [20,21]. This reduction manifests in attenuated metabolic pathways, diminished inflammatory responses, and impaired antigen presentation [22,23], collectively fostering an immunosuppressive “cold tumor” microenvironment that supports survival and therapeutic resistance [21,22,24–27]. On the other hand, biased redistribution of P-TEFb resources may enable preferential activation of proliferative networks, such as those driving clonal expansion and therapy resistance [9,12,13,15,28]. This hypothesized “global repression-local activation” paradigm has remained largely speculative, leaving its role in tumor evolution and clinical resistance unresolved.

In this study, we set out to investigate this paradigm and dissect the role of 7SK in colorectal cancer (CRC) chemotherapy resistance. We combined single-cell multi-omics profiling of longitudinal patient samples with functional perturbation to systematically unravel 7SK’s mechanism. We demonstrate that 7SK promotes resistance through a coherent dual-axis strategy: locally, it activates the JUN network to enhance proliferative capacity; globally, it suppresses transcriptional entropy to reshape the tumor microenvironment into an immunologically “cold” state. Furthermore, pan-cancer analyses confirm that this dual function of 7SK is a conserved feature across malignancies, underscoring its potential as a universal therapeutic target. Together, our work redefines 7SK from a static repressor to a dynamic transcriptional orchestrator in cancer, providing a new framework for ncRNA-mediated resistance and therapeutic intervention.

## Results

### Dynamic expansion of resistant clones under chemotherapy and the identification of a recurrent 7SK-high subpopulation

**Fig 1:**
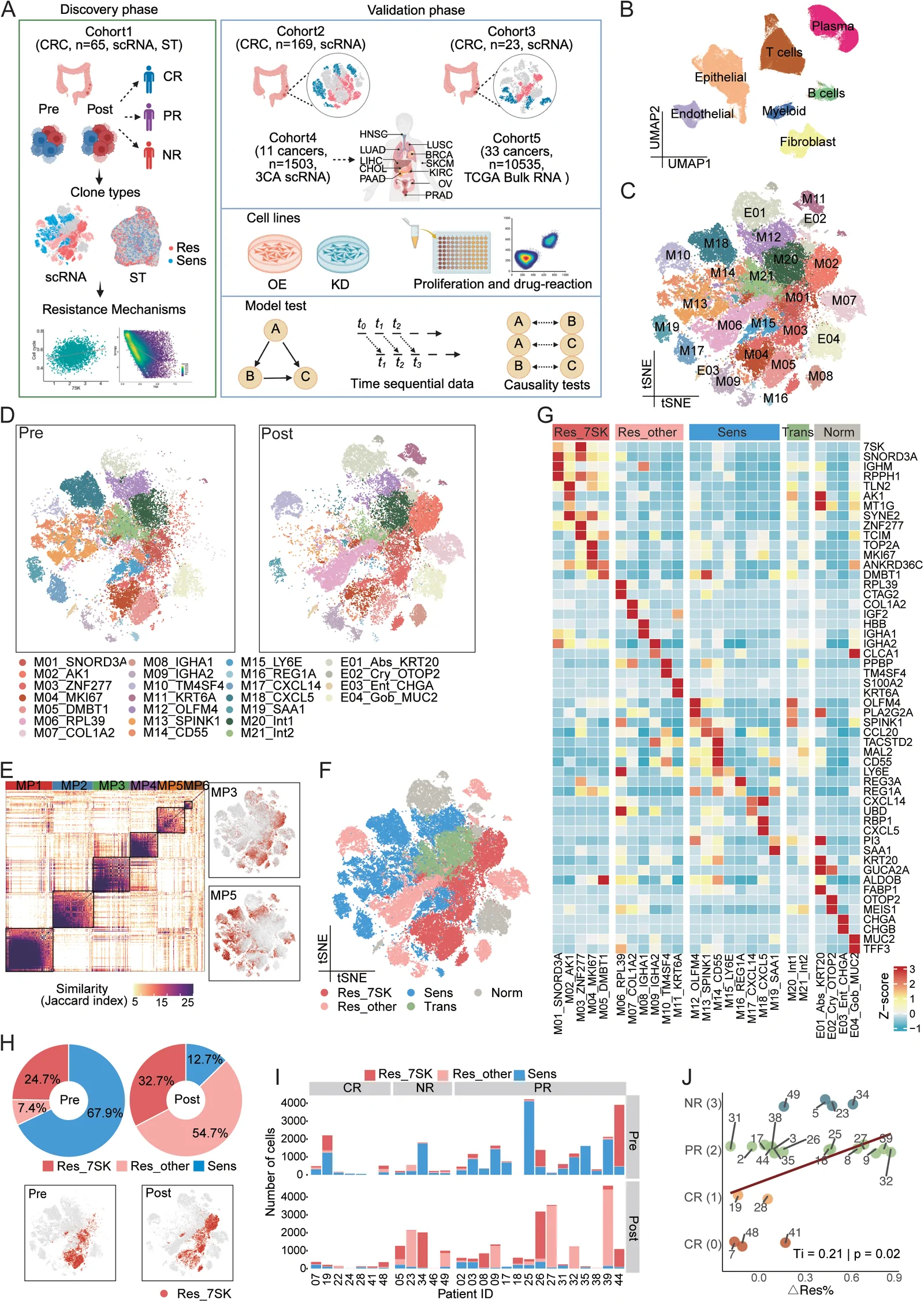
Identification of chemoresistant tumor clones and their clinical relevance in colorectal cancer. A. Study design schematic showing two phases: Cohort1 for discovery (29 CRC patients, scRNA-seq and Stereo-seq data) and Cohorts2-5 for validation and pan-cancer analysis. To further dissect the regulations suggested by the discovery analyses, *in vitro* experiments were performed using human colorectal cancer cell lines HCT-15 and LS180, and the Granger causality test along with transfer entropy inference were applied to infer causality and directionality of the identified regulatory signals. This figure was created in BioRender. Han, L. (2026) https://BioRender.com/d9gyesl.B. UMAP plot of 332,564 filtered cells annotated into epithelial, immune, endothelial, and stromal compartments.C. Clustering of tumor epithelial cells revealed 25 populations, including 19 malignant, 2 transitional and 4 normal-like epithelial lineages.D. Dynamic shifts in tumor epithelial populations before and after chemotherapy, highlighting enrichment of resistant clones of post-treatment.E. NMF decomposition of all epithelial transcriptomes identified six reproducible expression modules (MP1-MP6). Module activity patterns showed MP3 enrichment in resistant clones and MP5 enrichment in sensitive clones.F. Integrated classification defined four tumor epithelial categories: drug-sensitive (Sens), 7SK-high resistant (Res_7SK), sample-specific resistant (Res_other), and transitional subclones (Trans), along with normal-like epithelial cells.G. Heatmap displaying feature genes of tumor epithelial populations.H. Quantification of Res_7SK and Sens proportions pre- versus post-treatment, showing expansion of resistant populations.I. Clone distribution across clinical response groups; resistant clones predominated in non-responders, while sensitive clones dominated complete responders.J. Scatter plot of ΔRes% versus tumor regression grade, showing positive correlation between resistant clone expansion and poor outcome. ΔRes% defined as the proportion change of resistant clones (both Res_7SK and Res_other) after treatment compared with before treatment. 24 out of 27 patients with paired pre- and post-treatment samples were used in analysis (3 patients were filtered because of lacking enough tumor cells, see supplemental S1H Fig).

Transcriptomic heterogeneity underlies divergent responses to chemotherapy in colorectal cancer (CRC) [29,30]. To address this, we designed a two-phase study (Fig 1A, S1 Table): (1) Mechanism discovery using Cohort1 (29 pMMR/MSS CRC patients, from which there are 61 scRNA-seq samples including 54 paired pre-/post-treatment samples, 2 post-treatment samples, and 5 adjacent normal samples, and 4 post-treatment spatial transcriptomic profiling samples), stratified by clinical efficacy (CR/PR/NR) (S2 Table); (2) Validation and pan-cancer extension using Cohort2–5 (192 CRC scRNA-seq samples and 1,503 scRNA-seq samples from 11 cancer types, supplemented with 10,535 TCGA pan-cancer transcriptomes) to assess generalizability. This multi-cohort framework ensured both clinical relevance and cross-cancer validation.

Unsupervised clustering of 332,564 filtered cells revealed all major CRC tissue cell types (epithelial, T, myeloid, B, plasma, endothelial, and fibroblast) (Fig 1B). Within tumor epithelial cells, we resolved 19 malignant, 2 transitional and 4 normal-like subpopulations (Fig 1C, 1D), confirmed by their copy-number variation (CNV) patterns (S1A Fig). Comparison of pre-/post-treatment epithelial cells showed distinct clonal dynamics in response to chemotherapy, reflected in cell numbers and subtype composition (Figs 1D, S1B). Therefore, malignant cells were identified as resistant clones and sensitive clones according to their dynamics in response the treatment.

To further investigate their common molecular features among all subclones, we grouped expression profiles into six functional modules (MP1-MP6) via non-negative matrix factorization (NMF) (Fig 1E). Resistant and sensitive clones exhibited divergent module distributions: MP3 was enriched in resistant subclones, MP5 in sensitive cells (Fig 1E), and MP1 was broadly expressed across most subsets, whereas MP2 showed higher expression in normal-like subpopulations (S1C- S1D Fig). Finally, integrating clustering, dynamic treatment shifts, expression programs, and also considering the cross-sample recurrence (S1D - S1E Fig), tumor epithelial cells were categorized into five classes: drug-sensitive (Sens), 7SK-high drug-resistant (Res_7SK), sample-specific resistant (Res_other), transitional subclones (Trans), and normal-like epithelial cells (Norm) (Fig 1F). Notably, different subpopulations within the Res_7SK group shared a common expression pattern characterized by elevated 7SK, SNORD3A, RPPH1, and SYNE2 expression (Fig 1G).

Quantitative analysis highlighted expansion of the Res_7SK population, rising from 24.7% pre-treatment to 32.7% post-treatment (p < 0.001), while Sens cells declined from 67.9% to 12.7% (p < 0.001) (Fig 1H). Clonal distributions also stratified patient outcomes. CR samples were enriched for sensitive cells, whereas after treatment, the number of resistant cells in NR samples was much higher than that in CR samples (Fig 1I). Importantly, the dynamic increase in resistant clones (ΔRes%) significantly correlated with tumor regression grade (TRG) (p = 0.02) (Fig 1J).

Together, we mapped the landscape of chemoresistant clones in CRC and identified a recurrent 7SK-high resistant population (Res_7SK) that exhibits distinct molecular features and cross-sample reproducibility. While the overall expansion of resistant clones correlates with clinical outcome, Res_7SK distinguishes itself as a reproducible and transcriptionally defined state, warranting further mechanistic investigation.

### The Res_7SK subclone is defined by 7SK overexpression, hyperactivated proliferation, and a structured JUN transcriptional network

Having identified Res_7SK as a clinically relevant resistant population, we next sought to define its core molecular features. Multi-dimensional analysis revealed three defining characteristics: specific upregulation of lncRNA 7SK, hyperactivation of mitotic pathways, and engagement of the JUN-centered transcriptional network.

**Fig 2:**
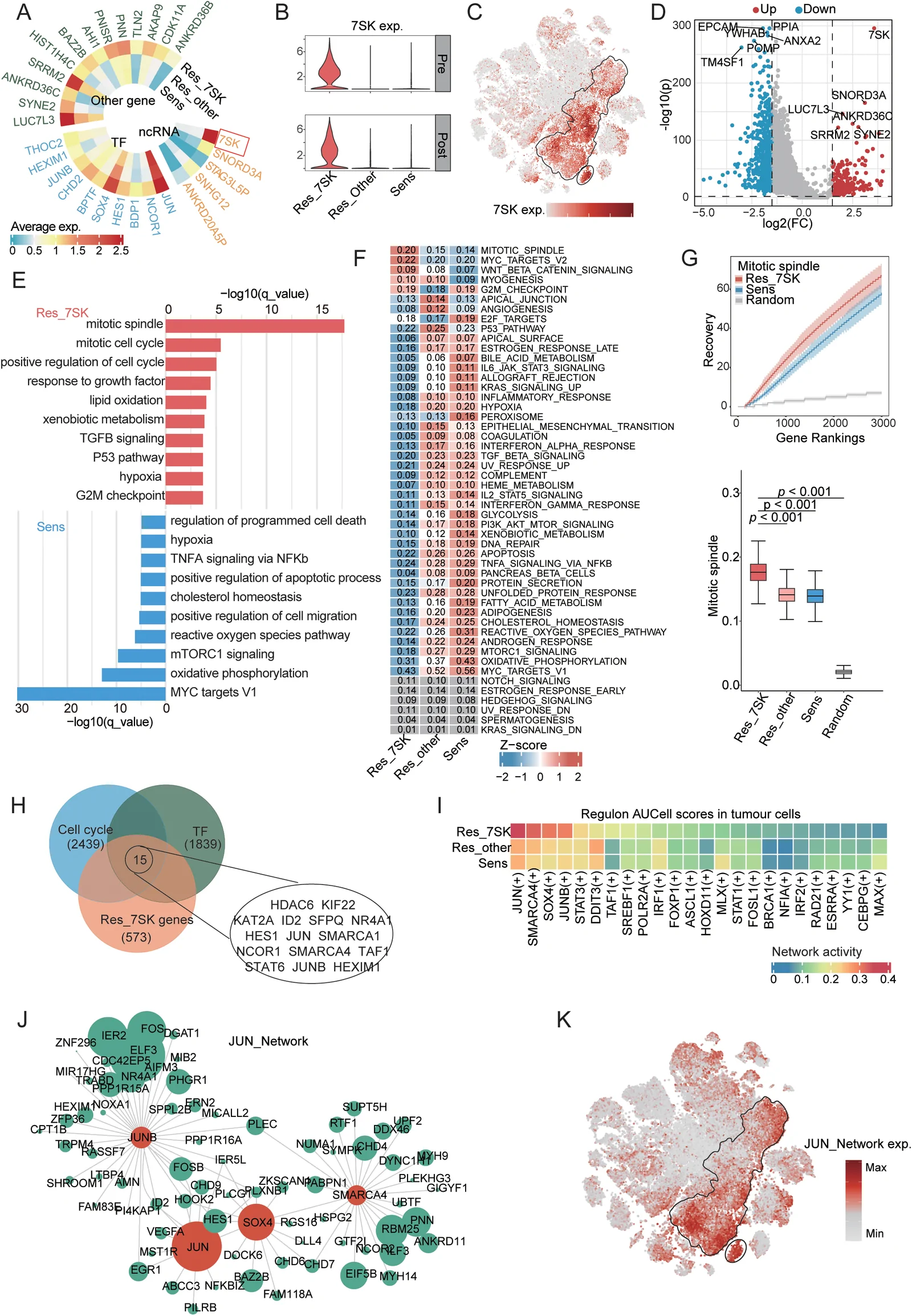
7SK expression and cell proliferation are specifically upregulated in chemoresistant subclones. A. Heatmap of Res_7SK signature genes identified by differential expression analysis (Res_7SK versus Res_other and Sens; p < 0.05, log_2_FC > 1.5), with 7SK shown as the top upregulated ncRNA.B. Violin plots showing 7SK expression in Res_7SK and other subclones (before and after treatment), demonstrating specific upregulation in Res_7SK.C. t‑SNE feature plot of 7SK expression, with Res_7SK cells exhibiting elevated levels. Res_7SK cells outlined by black line. Mean expression: Res_7SK = 2.22, Res_other = 0.47, Sens = 0.46; p < 0.001 for Res_7SK vs. Res_other and Sens.D. Volcano plot of differentially expressed genes comparing Res_7SK with Sens and Res_Other, highlighting 7SK as the most significantly upregulated gene.E. GO enrichment analysis showing Res_7SK genes linked to mitosis, while Sens genes were enriched for MYC pathway and oxidative metabolism.F. GSEA enrichment confirming strong mitotic spindle activation in Res_7SK. Enrichment scores are presented for each gene set of subclones. Scores with no difference among subclones are colored with grey.G. Enrichment curves and box plots of mitotic spindle scores across subclones, showing significant elevation in Res_7SK.H. Res_7SK specific genes and TF regulators are enriched in cell cycle function.Venn diagram of Res_7SK-specific genes (lower circle; Res_7SK vs. Res_other and Sens, p < 0.05, n=573), cell cycle-related genes (upper-left circle, n= 2439), and transcription factors (TF, upper-right circle, n=1839). The triple intersection contains 15 genes, listed on the right.I. TF-Network reconstruction showing JUN regulatory activation in Res_7SK.J. Graph representation of JUN regulatory network, with JUN as a central hub.K. Preferential activation of JUN network activity in Res_7SK cells. Res_7SK cells outlined by black line. Mean score: Res_7SK = 0.80, Res_other = 0.56, Sens = 0.54; p < 0.001 for Res_7SK vs. Res_other and Sens.

Res_7SK clones displayed significant overexpression of regulatory genes, including non-coding RNAs (7SK, SNORD3A), transcription factors (BDP1, JUN, HES1, HEXIM1, CHD2), and chromatin regulators (ANKRD36C, HIST1H4C) (Fig 2A). Among these, 7SK exhibited the highest expression and strongest inter-clonal specificity, establishing it as a core marker of Res_7SK. Elevated 7SK expression was consistently observed before and after treatment, distinguishing Res_7SK from other subclones (Fig 2B), with cell distribution in tSNE embedding space confirming its localization within resistant compartments (mean expression: Res_7SK = 2.22, Res_other = 0.47, Sens = 0.46; p < 0.001 for Res_7SK vs. Res_other and Sens) (Fig 2C). Comparative analysis of Res_7SK versus Sens clones and other resistant clones further identified 7SK as the most subtype-specific differentially expressed gene (Fig 2D). Modules of expression programs confirmed 7SK as a strong character of MP3, which module represents the Res_7SK subclones (S2A Fig).

Functional annotation revealed that Res_7SK signature genes were enriched in mitotic processes (spindle assembly, cell cycle regulation), whereas Sens signature genes were enriched in MYC and metabolic pathways (oxidative phosphorylation, glycolysis) (Figs 2E, S2B). Gene set enrichment analysis confirmed stronger activation of the mitotic spindle pathway in Res_7SK (enrichment score = 0.20 vs. 0.14, p < 0.001, Wilcoxon test) (Fig 2F–2G). Importantly, no other hallmark pathways showed significant enrichment, underscoring a proliferation-dominant phenotype.

To pinpoint the transcriptional drivers of this proliferative phenotype, we reconstructed gene regulatory networks. Among 573 differentially expressed genes, 15 transcription factors were directly linked to cell cycle regulation, including JUN, HES1, ID2, SMARCA4, and JUNB (Fig 2H). These factors collectively enhanced proliferation through chromatin remodeling (SMARCA4), cell cycle promotion (ID2), and mitotic progression (JUN/JUNB). Network analysis identified a TF module comprising JUN, SMARCA4, SOX4, and JUNB, with JUN showing maximal network activity (Fig 2I). This module regulated mitotic effectors while dampening pro-apoptotic signaling (P53 pathway) (S2D Fig). Network topology of these four active TF networks demonstrated that JUN acted as the central hub and connected with other TFs, (Fig 2J). The interconnected JUN, SOX4, HES1, and JUNB modules formed an integrated transcriptional system (JUN_network), with the whole network activity specifically elevated in Res_7SK cells (mean score: Res_7SK = 0.80, Res_other = 0.56, Sens = 0.54; p < 0.001 for Res_7SK vs. Res_other and Sens) (Fig 2K).

Collectively, these results establish a triad of features characterizing Res_7SK: specific 7SK overexpression, a hyperproliferative state, and structured activation of a JUN-centered regulatory network. This prompted us to investigate whether 7SK plays a causal role in regulating this network and driving the resistant phenotype.

### 7SK drives tumor proliferation and chemoresistance by directly activating the JUN network

**Fig 3:**
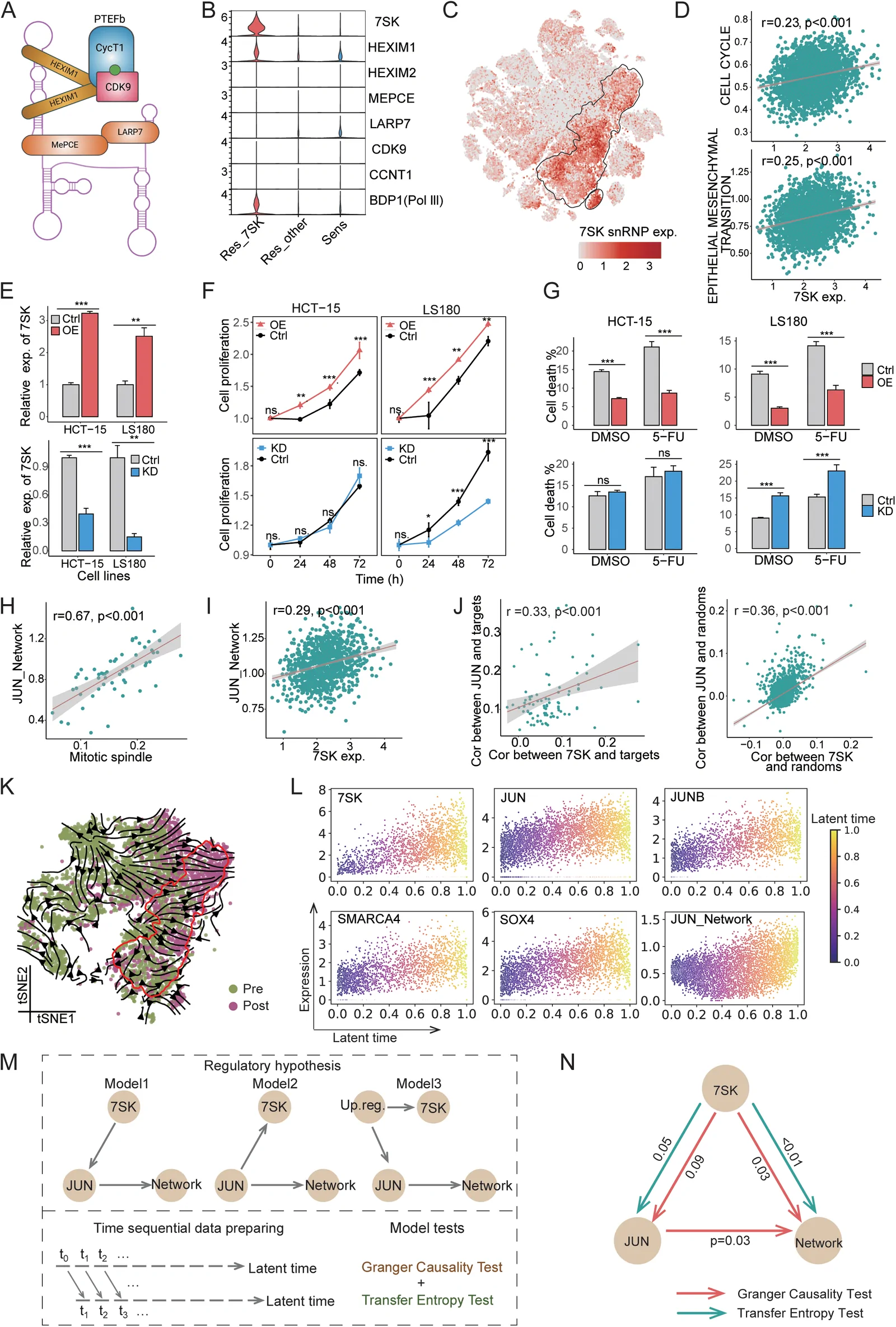
7SK promotes tumor cell proliferation and chemoresistance via the JUN network. A. Schematic of the canonical 7SK snRNP complex, including HEXIM1, MEPCE, and P-TEFb.B. Violin plots showing upregulation of 7SK, HEXIM1, and BDP1 in Res_7SK cells.C. t-SNE feature plot showing 7SK snRNP genes expression, with Res_7SK cells exhibiting elevated levels (the cells outlined by black line). Mean score: Res_7SK = 0.71, Res_other = 0.28, Sens = 0.31; p < 0.001 for Res_7SK vs. Res_other and Sens.D. Correlation scatter plots linking 7SK levels with cell cycle and proliferation pathway scores.E. Design and validation of 7SK overexpression and knockdown constructs in colorectal cancer cell lines. HCT-15 and LS180 cells were transfected with plasmid-based overexpression vectors or shRNA-expressing lentiviral constructs targeting 7SK. qRT-PCR confirmed significant upregulation of 7SK in the overexpression group, while shRNA-mediated knockdown efficiently reduced 7SK expression levels.F. Proliferation assays showing enhanced growth upon 7SK overexpression in CRC cell lines.G. Chemotherapy survival assays showing increased resistance upon 7SK overexpression and sensitization upon knockdown.H. Scatter plot showing positive correlation between JUN network activity and proliferation scores.I. Correlation plot showing positive association between 7SK expression and JUN network activity.J. Synergistic regulation of target genes by 7SK and JUN. Target genes are defined as the downstream genes of transcription factors identified in the network.K. Trajectory analysis reconstructed the evolution of tumor subclones before and after treatment, revealing a dynamic transition from Sens to Res_7SK cells. Res_7SK cells outlined by red line.L. Both 7SK and the JUN network exhibited dynamic changes along the inferred trajectory, indicating a strong association with tumor evolution.M. Regulatory models and tests. We proposed three potential regulatory models: Model 1(7SK regulates the JUN network), Model 2(JUN regulates 7SK), and Model 3 (A common upstream regulator controls both 7SK and JUN). These models were tested using Granger Causality Test and transfer entropy inference.N. Model evaluation supported the hypothesis that 7SK regulates the JUN network. Both Granger causality and transfer entropy tests revealed a significant unidirectional causal relationship or information flow from 7SK to JUN, providing evidence in favor of Model 1.

To establish a causal link between 7SK and the observed phenotypes, we turned to functional assays. The canonical 7SK snRNP complex consists of 7SK ncRNA, HEXIM1, MEPCE, LARP7, and P-TEFb (CDK9/CCNT1) [10,15], which modulates RNA polymerase II elongation (Fig 3A). In Res_7SK clones, expression of 7SK, HEXIM1, and BDP1 (subunit of RNA polymerase III transcription factor) was significantly elevated compared with other subclones (Fig 3B). Cells highly expressing snRNP complex genes were concentrated in Res_7SK subpopulations (mean score: Res_7SK = 0.71, Res_other = 0.28, Sens = 0.31; p < 0.001 for Res_7SK vs. Res_other and Sens) (Fig 3C), suggesting selective reinforcement of transcriptional control in resistant clones. Correlation analysis confirmed that 7SK expression was positively associated with cell cycle (r = 0.23, p < 0.001) and proliferation pathways (r = 0.25, p < 0.001), but showed no significant correlation with other tumor hallmarks (Figs 3D, S3A).

Functional validation in CRC cell lines (HCT-15, LS180) demonstrated that 7SK overexpression significantly increased proliferation and conferred resistance to 5-FU, whereas its knockdown produced the opposite effects (Fig 3E–3G). Overexpression of 7SK significantly increased proliferation (MTS assay at 72h: HCT-15: 2.10 vs. 1.71, p < 0.001; LS180: 2.47 vs 2.21; p < 0.01), whereas knockdown reduced proliferation by 25.8% (MTS assay: HCT-15: not significant; LS180: 1.44 vs 1.94, p < 0.001) (Fig 3F). Under 5-FU treatment, 7SK-overexpressing cells showed lower mortality than controls (HCT-15: 8.6% vs. 21.1%, p < 0.001; LS180: 6.3% vs. 14.1%, p < 0.001), while knockdown significantly increased mortality (HCT-15: not significant; LS180: 23.0% vs. 15.3%, p < 0.001) (Figs 3G, S3B). These results demonstrate that 7SK directly enhances proliferative capacity and confers chemoresistance.

We then asked how 7SK expression relates to the JUN network activity. We found a strong positive correlation between 7SK expression and JUN_network activity. JUN_network activity was strongly correlated with cell proliferation as expected (r = 0.67, p < 0.001) (Fig 3H), and with 7SK expression (r = 0.29, p < 0.001) (Fig 3I), indicating a “7SK-JUN-proliferation” axis regulation. At the target-gene level, we evaluated whether 7SK and JUN showed coordinated associations with JUN_network targets by performing a gene-wise correlation analysis. For each gene in the JUN_network target set, we calculated its correlation with 7SK expression and, separately, its correlation with JUN expression. The two sets of correlation coefficients were significantly positively correlated (r = 0.33, p < 0.001), indicating that JUN_network targets more strongly associated with JUN also tended to show stronger association with 7SK. This supports coordinated regulation between 7SK and the JUN_network at the target-gene level (Fig 3J). Similarly, at the genome-wide level, we extended this analysis to random/background genes and observed a strong positive association between JUN-gene correlations and 7SK-gene correlations (r = 0.36, p < 0.001). These results indicate that 7SK-mediated expression patterns are concordant with JUN-associated gene regulation, supporting a model in which 7SK potentiates JUN transcription factor activity to sustain proliferative and resistant phenotypes. Trajectory analysis reconstructed the evolutionary path of Res_7SK and Sens cells across treatment, revealing a continuous transition from drug-sensitive to resistant states (Fig 3K). Along this inferred trajectory, both expression level of 7SK and JUN network show trends of dynamic upregulation, suggesting that their activation is closely associated with tumor clonal evolution (Fig 3L).

To rigorously determine the direction of regulation, we employed Granger causality and transfer entropy analyses. These tests unanimously supported a unidirectional causal flow from 7SK to JUN activity, rather than the reverse. We formulated three potential models: Model 1, 7SK regulates the JUN network; Model 2, JUN regulates 7SK; and Model 3, a common upstream factor (e.g., RNA polymerase III related genes or transcription regulation factors, including TFIIIA, TFIIIB, TFIIIC, BDP1, etc.) simultaneously regulates the expression of both 7SK and JUN (Fig 3M). Using Granger causality analysis and transfer entropy inference, we quantitatively tested these hypotheses. The results strongly supported Model 1, demonstrating a unidirectional causal flow from 7SK to JUN activity rather than the reverse (Figs 3N, S3C - S3D). These findings provide robust statistical and information-theoretic evidence that 7SK directly regulates JUN network activation, thereby promoting proliferative signaling and chemoresistance.

Thus, we demonstrate that 7SK is not merely a marker but a causal upstream regulator that promotes proliferation and chemoresistance by activating the JUN transcriptional network.

### 7SK suppresses transcriptional entropy and fosters an immunosuppressive microenvironment

**Fig 4:**
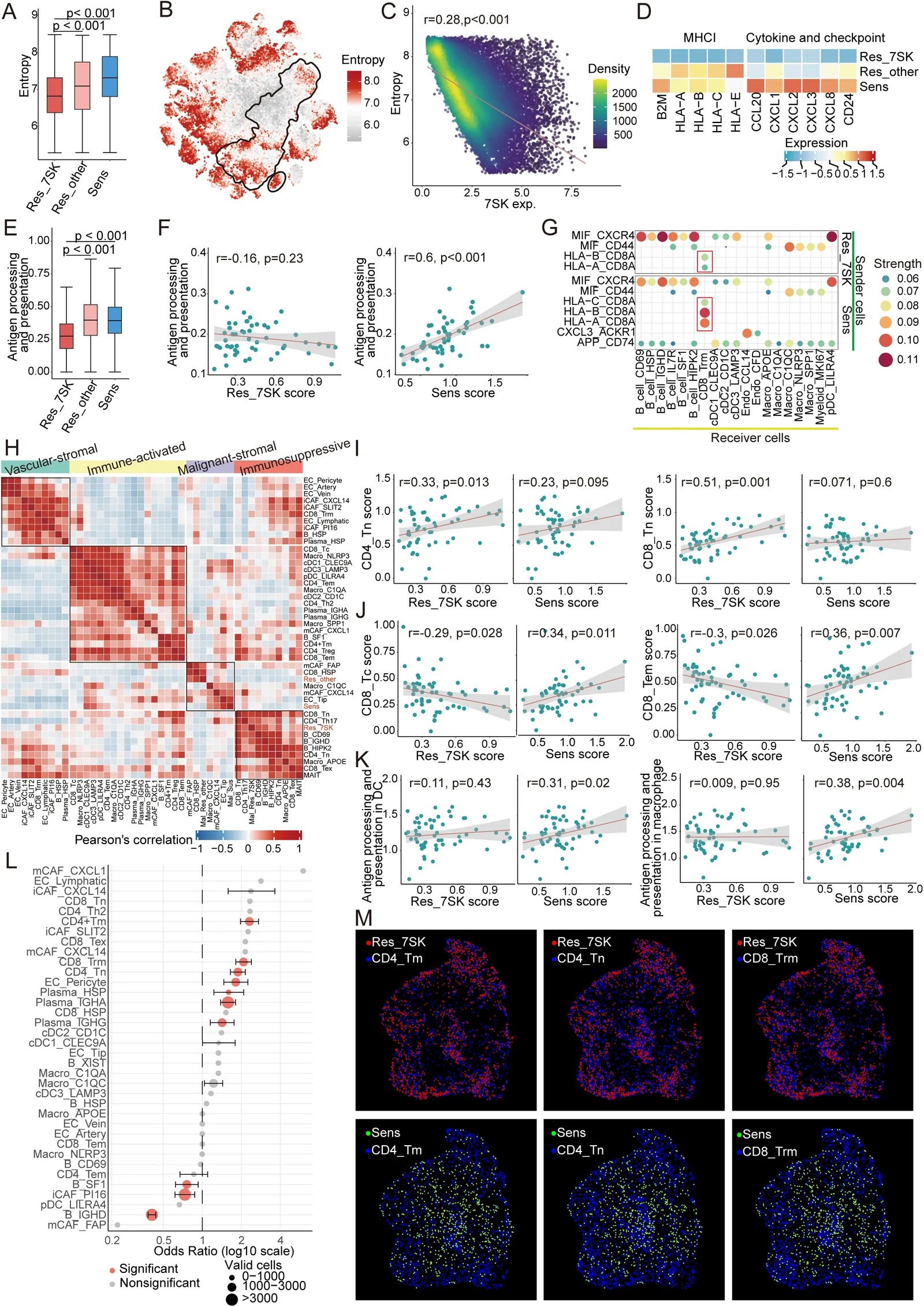
7SK reduces transcriptional entropy and establishes an immunosuppressive tumor microenvironment. A. Box plots of transcriptional entropy across tumor epithelial subtypes, showing lowest entropy in Res_7SK.B. Entropy maps of t-SNE embedding showing entropy reduction in Res_7SK subclones. Res_7SK cells outlined by black line. Mean entropy: Res_7SK = 6.82, Res_other = 7.09, Sens = 7.32; p < 0.001 for Res_7SK vs. Res_other and Sens.C. Scatter plot showing strong negative correlation between 7SK expression and transcriptional entropy.D. Heatmap showing reduced expression of chemokines and MHC-I genes in Res_7SK.E. Pathway activity plots showing impaired antigen presentation in Res_7SK.F. Res_7SK did not show a correlation with HLA-mediated antigen presentation, whereas the Sens subclone displayed a significant positive correlation.G. Cell-cell interaction analysis revealed that tumor-T interactions mediated by MHC I molecules were markedly weaker in Res_7SK than in Sens. This finding suggests that tumor cells in the Res_7SK group have a significantly reduced ability to present antigens to CD8⁺ T cells via HLA molecules, thereby dampening the anti-tumor immune response.H. Cellular modules highlighting enrichment of immunosuppressive populations with Res_7SK. Res_7SK cells were associated with an "immunosuppressive module" containing exhausted T cells (CD8_Tex), naive T cells (CD4_Tn, CD8_Tn), regulatory T cells (Treg). Tumor epithelial subtypes Res_7SK, Res_other, Sens were colored in red.I. Correlation plots showing positive association between Res_7SK and naive T cells.J. Correlation plots showing negative association between Res_7SK and effector T cells, while Sens positively associated with effector T cells.K. Scatter plot showing absence of correlation between Res_7SK and myeloid antigen presentation, while significantly positive correlations in Sens.L. Spatial colocalization confirming the adjacency of Res_7SK and inactive T cells.The plot shows the spatial co-localization enrichment around Res_7SK cells compared to Sens cells in sample ST-NR1. Odds ratios (OR, displayed on a log10 scale) were calculated using Fisher’s exact test based on 2×2 contingency tables, comparing the proportions of each cell type co-localized around Res_7SK cells versus sensitive cells. An OR > 1 indicates that the cell type preferentially co-localizes with Res_7SK cells, whereas an OR < 1 indicates preferential association with sensitive cells. Error bars represent 95% confidence intervals. Point colors indicate statistical significance (red: p < 0.05; gray: not significant). Point sizes reflect the effective number of cells used in the analysis.M. Res_7SK tumor cells were spatially co-localized with inactive T cells (naïve T, Trm, and Tm), a pattern that was markedly distinct from that observed in Sens.

Beyond driving proliferation, we hypothesized that 7SK’s canonical repressive function might concurrently shape the tumor microenvironment to facilitate immune escape. Indeed, transcriptional entropy analysis showed that Res_7SK clones possess significantly lower entropy than other subclones, reflecting a constrained transcriptional state (mean entropy: Res_7SK = 6.82, Res_other = 7.09, Sens = 7.32; p < 0.001 for Res_7SK vs. Res_other and Sens) (Fig 4A–4B). Importantly, 7SK expression negatively correlated with entropy (p < 0.001), indicating that 7SK limits intratumoral heterogeneity by globally repressing transcriptional activity (Fig 4C), consistent with previous reports that 7SK was recognized as a global transcriptional repressor that inhibits transcription elongation by sequestering P-TEFb [4–10].

Consistent with a state of broad transcriptional suppression, Res_7SK clones exhibited marked downregulation of antigen presentation machinery and key chemokines (Fig 4D–4E). Chemokines (CCL20, CXCL1, CXCL8) and MHC class I molecules (HLA-A, HLA-B, B2M) were downregulated, leading to impaired antigen presentation and weakened T cell activation. At the sample level, Res_7SK signatures correlated negatively with antigen presentation activity, whereas Sens signatures correlated positively (Fig 4F), indicating 7SK suppresses immune recognition pathways.

This transcriptional quiescence translated into altered cellular crosstalk. We identified the subtypes of B, Plasma, CD8 + T, CD4 + T, myeloid, endothelial, and fibroblast cells in the tumor microenvironment (S4A and S4B Fig), to study their communication and coordination with tumor cells. Cell-cell interaction mapping revealed diminished MHC I mediated antigen presentation from Res_7SK to CD8 + T cells (Fig 4G). Specifically, the HLA-CD8A crosstalk signals between Res_7SK and CD8_Trm cells were weaker than Sens. According to the cellular coordination, tumor cells and cells from microenvironment were partitioned into an “immunosuppressive module” (exhausted CD8_Tex, naïve T cells, Tregs and memory T cells) and an “effector module” (cytotoxic T cells, myeloid cells, plasma cells), with Res_7SK preferentially associating with the former. (Fig 4H). Immune cell correlation analysis (Fig 4I–4K) provided mechanistic detail: Res_7SK expression correlated positively with naïve T cells (CD8_Tn r = 0.51 p < 0.001; CD4_Tn r = 0.33; p = 0.013) (Fig 4I) and negatively with effector T subsets (CD8_Tc r = -0.29, p = 0.028; CD8_Tem r = -0.30, p = 0.026) (Figs 4J and S4C). Notably, Res_7SK showed no association with myeloid antigen presentation (Fig 4K), underscoring its immunosuppressive nature. In contrast, Sens signatures correlated positively with effector T cells and myeloid antigen-presenting cells (macrophages r = 0.38, p = 0.004; dendritic cells r = 0.31; p = 0.02). Spatial transcriptomics validation directly demonstrated the preferential co-localization of Res_7SK cells with inactive T cells (CD4 + Tn OR=1.88; CD8 + Trm OR=2.08) (Figs 4L–4M, S4D). This segregation of immunosuppressive versus effector T cell niches establishes localized immune tolerance.

In essence, 7SK engenders an immunologically “cold” tumor by globally repressing transcriptional activity, thereby dampening antigen presentation and promoting a localized immunosuppressive niche.

### Pan-cancer analyses validate the universal role of 7SK in coordinating proliferation and transcriptional repression

**Fig 5:**
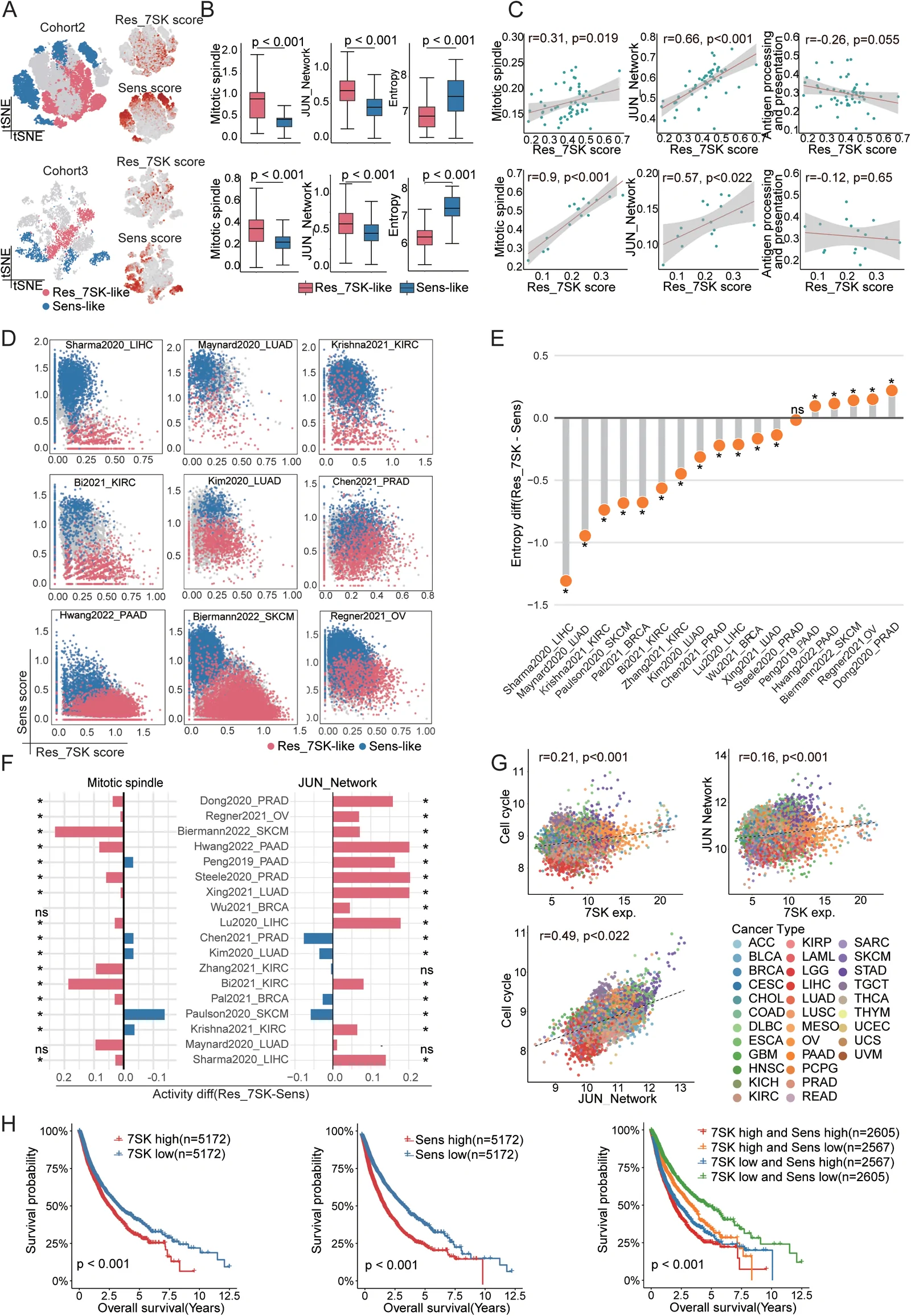
Pan-cancer validation of 7SK’s dual-axis role. A. t-SNE plots from Cohorts2-3 scRNA confirming the recurrence of Res_7SK-like and Sens-like subclones in independent CRC samples.B. Box plots comparing transcriptional entropy and proliferation between Res_7SK-like and Sens-like clones in validation cohorts.C. Scatter plots showing pathway correlations with Res_7SK positively linked to proliferation, negatively to antigen presentation.D. Recurrence of Res_7SK-like and Sens-like clones across malignancies of multiple cancer types from Cohort4.E. Box plots showing lower entropy of Res_7SK compared to Sens across cancers.F. JUN_network and Proliferation scores across cancers showing Res_7SK consistently enriched for cell cycle activity.G. TCGA pan-cancer scatter plots showing 7SK positively correlated with proliferation and JUN activity.H. Kaplan-Meier curves showing poor overall survival in patients with high 7SK expression.

Finally, we assessed the generalizability of this 7SK-mediated dual-axis mechanism across human cancers. In two independent colorectal cancer single-cell validation cohorts (Cohort2 and Cohort3), Res_7SK and Sens subpopulations were robustly recapitulated (Fig 5A). Subtype-specific gene expression profiles enabled robust separation in t-SNE space, with Res_7SK clones occupying transcriptional territories different from Sens clones, consistent with their molecular heterogeneity. This spatial segregation faithfully recapitulated findings from the discovery cohort, validating Res_7SK as a reproducible resistant subpopulation.

Within these validation cohorts, Res_7SK clones consistently exhibited reduced transcriptional entropy compared to Sens clones (p < 0.001) while displaying elevated mitotic spindle and JUN_network activity (p < 0.001; Fig 5B). Correlation analysis further confirmed that Res_7SK signature scores positively associated with proliferative pathways and negatively with antigen presentation activity (Fig 5C), supporting the mechanism whereby 7SK simultaneously drives proliferation and immune suppression, establishing a transcriptionally constrained yet proliferative “cold tumor” state.

Strikingly, Res_7SK-like subpopulations were identified across multiple cancer types in a pan-cancer single-cell dataset (Cohort4) (Fig 5D). Across these diverse malignancies, Res_7SK clones exhibited lower transcriptional entropy (Fig 5E), and higher JUN_network activities (Fig 5F) than Sens clones in multiple cancer types, including LIHC, LUAD, KIRC, and BRCA. However, opposite patterns were observed in some tumor types, including PAAD and OV, whereas SKCM and PRAD showed heterogeneous patterns. TCGA pan-cancer analysis (Cohort5) further confirmed these mechanistic links. 7SK expression positively correlated with cell cycle activity (r = 0.21, p < 0.001) and JUN_network activation (r = 0.16, p < 0.001), while JUN_network strongly correlated with cell cycle signaling (r = 0.49, p < 0.001) (Fig 5G). These results highlight a conserved 7SK-JUN regulation driving proliferation across cancers, although correlation strength varied by tumor type, suggesting context-dependent modulation. Clinically, high 7SK expression predicted significantly worse overall survival across cancer types (p < 0.001; Fig 5H). This establishes 7SK not only as a driver of proliferation and immune escape but also as a poor prognostic biomarker with translational potential for therapeutic targeting.

Therefore, the dual role of 7SK, orchestrating local oncogenic activation via JUN while enforcing global transcriptional repression, is a conserved mechanism across cancers, underpinning its utility as a prognostic biomarker and a compelling pan-cancer therapeutic target.

In summary, our work resolves the longstanding paradox of 7SK function in cancer by demonstrating that its dual role is not contradictory but cooperative. We provide direct evidence for the hypothesized “global repression-local activation” paradigm, showing how 7SK drives chemotherapy resistance by locally licensing proliferative dominance through JUN network activation, while globally enforcing an immunologically cold state through transcriptional entropy suppression. By establishing this unified model and confirming its conservation across cancers, we position the 7SK-JUN axis not merely as a driver of resistance but as a promising therapeutic frontier for overcoming both chemoresistance and immune evasion.

## Discussion

In this study, we systematically uncovered the pivotal role of the ncRNA 7SK in driving chemotherapy resistance in colorectal cancer (CRC) using single-cell multi-omics analysis coupled with functional validation. We demonstrate that 7SK confers resistance through a dual-axis regulatory mechanism: (i) preferential activation of the JUN transcriptional network, thereby enhancing tumor cell proliferation; and (ii) global reduction of transcriptional entropy, which suppresses antigen presentation and reshapes the tumor microenvironment toward an immunosuppressive, “cold” state. These findings redefine 7SK from its conventional designation as a global transcriptional repressor and instead highlight its dynamic and context-dependent role in balancing local transcriptional activation with global suppression. Importantly, pan-cancer analyses confirmed the conservation of this mechanism across multiple malignancies, suggesting that 7SK represents a universal driver of tumor proliferation, immune evasion, and poor prognosis, as well as a promising therapeutic target.

Our work provides a conceptual framework to resolve the apparent paradox of how 7SK can simultaneously function as a global repressor and a local activator. We propose an integrative model wherein the overarching upregulation of 7SK in resistant clones reinforces its canonical role by augmenting the 7SK snRNP complex, leading to pervasive P-TEFb sequestration. This accounts for the observed global diminishment of transcriptional entropy and the consequent downregulation of immunogenic signals, such as antigen presentation and chemokine pathways. However, contrary to the classical model, our data indicate that this repressive state is not absolute. We posit that within this transcriptionally constrained landscape, Res_7SK clones have co-opted a mechanism for the biased, local mobilization of P-TEFb resources. This model is strongly supported by our causal inference analyses, which pinpoint 7SK as the upstream driver of JUN network activation. While the precise signal that triggers the localized release of P-TEFb at specific genomic loci (e.g., JUN and its targets) remains a key open question, it is plausible that tumor-specific stressors or co-factors orchestrate a contextual remodeling of the 7SK snRNP, thereby permitting selective transcriptional bursting of pro-survival genes. This paradigm of “focused escape from global repression” elegantly reconciles the dual transcriptional outputs we report and underscores 7SK’s role as a key regulator of transcriptional resource allocation in cancer cells.

Despite these insights, this study has limitations that also chart the course for future inquiry. While our functional assays conclusively established 7SK’s role in promoting proliferation and chemoresistance, and our computational approaches robustly supported a unidirectional regulatory flow from 7SK to JUN, the direct biochemical mechanism of this activation, such as demonstrating the localized dissociation of the 7SK snRNP at JUN-associated cis-regulatory elements, awaits further dissection using techniques like ChIRP-MS or chromatin-bound proteomics. Furthermore, the very principle of selectivity, how the cell dictates which genes remain suppressed and which are selectively activated, represents the central fascinating mystery emerging from our work. We hypothesize that interactions with tissue-specific transcription factors or epigenetic writers may guide the spatial patterning of P-TEFb availability. Systematic mapping of these interactions will be essential to fully elucidate the rules of 7SK-mediated gene control. Finally, broader validation of this 7SK-driven resistance program in vivo, using genetically engineered organoid or animal models, will be crucial to firmly establish its therapeutic tractability.

Given the inherent technical and biological variability in single-cell transcriptomic datasets, we carefully evaluated whether our conclusions were influenced by data quality heterogeneity. To this end, we repeated all major analyses after applying a stringent quality filter (>2,000 reads per cell), which retained 42,695 high-quality cells for downstream analyses (S6A Fig). The Res_7SK population was robustly identified with 7SK remaining the most prominent marker (S6B Fig), and all key pathway associations including JUN network activation, enhanced proliferation, and reduced transcriptional entropy were fully recapitulated (S6C Fig). The consistency between the original and stringently filtered datasets, together with the reproducibility of our findings across multiple independent cohorts, collectively argues against the possibility that our conclusions are artifacts of data quality variation.

Biological sex represents an important consideration in tumor transcriptomic analyses. Given the higher proportion of female samples in our discovery cohort and the observed overexpression of XIST in Res_7SK population, we evaluated whether the Res_7SK program might be confounded by sex-associated transcriptional differences. 7SK enrichment in Res_7SK cells was observed in both female and male patients (S6D Fig). Sex-stratified analyses independently identified the Res_7SK population in both sexes (S6E Fig), with 220 marker genes overlapping between female- and male-derived populations, including core genes such as 7SK, ZNF277, SYNE2, JUN, JUNB, HES1, SNORD3A, and HEXIM1 (S6F Fig). The functional features including JUN network activation, enhanced proliferation, and reduced transcriptional entropy were consistently observed across sexes (S6G Fig). To minimize potential confounding, sex-associated genes were excluded from the core signature in our study. These results confirm that the Res_7SK program represents a sex-independent transcriptional state.

Notwithstanding these limitations and concerns, our findings provide a new advance in understanding ncRNA-mediated tumor resistance. By defining the 7SK-centered dual-axis paradigm, we uncover how a single ncRNA can function as a molecular toggle, simultaneously reinforcing proliferative signaling and suppressing immunogenicity to orchestrate clonal evolution under therapeutic pressure. Beyond mechanistic insights, this work also illustrates the power of single-cell multi-omics to resolve complex transcriptional governance at fine resolution. Future research should build upon this framework to: (i) decrypt the molecular code governing 7SK’s target selectivity across diverse cancers; (ii) visualize the dynamic interplay between 7SK and the transcriptional machinery in real time; and (iii) pioneer therapeutic strategies that disrupt the 7SK-JUN axis to re-sensitize tumors to treatment. Collectively, these efforts may pave the way for novel therapies that simultaneously target tumor proliferation and immune evasion, offering new hope for patients with chemotherapy-refractory cancers.

## Materials and methods

### Data collection

This study consisted of two major components: (i) discovery of drug resistance mechanisms in colorectal cancer (CRC), and (ii) validation and paradigm investigation across independent cohorts and pan-cancer datasets. We collected five cohorts (Cohorts 1–5, S1 Table) to support this analysis.

### Cohort 1 (Discovery cohort)

Cohort1 was used for mechanism discovery and included 61 single-cell RNA-seq (scRNA-seq) samples and 4 spatial transcriptome (Stereo-seq) samples derived from 29 pMMR/MSS colorectal cancer patients treated with neoadjuvant chemotherapy (CNGBdb accession: CNP0004138) [31]. A total of 54 paired tumor samples were collected before and after treatment, 2 samples were collected after treatment, and 5 adjacent normal samples from 5 of 29 patients. Based on tumor regression grade (TRG), 27 patients were classified into three groups: complete response (CR, n = 7), partial response (PR, n = 15), and no response (NR, n = 5). The remaining 2 patients were not evaluable for response. Additionally, 4 post-treatment samples were subjected to Stereo-seq spatial transcriptomics (ST) profiling.

### Cohort 2 and Cohort 3 (CRC validation cohorts)

Cohort2 comprised single-cell transcriptome data of 169 samples from 22 MSI-H/MSS colorectal cancer patients (GEO accession: GSE236581) [24]. Cohort3 consisted of 23 single-cell transcriptomes from 16 MSI-H/MSS colorectal cancer patients (GEO accession: GSE200997) [32]. These two cohorts served as independent validation sets for the signals and mechanisms discovered in Cohort1.

### Cohort 4 (Pan-cancer single-cell dataset)

Cohort4 included 1503 single-cell transcriptomes spanning 11 cancer types, obtained from the 3CA database (https://www.weizmann.ac.il/sites/3CA/) [33]. This cohort was used to explore the universality of 7SK-mediated regulatory mechanisms across cancers.

### Cohort 5 (TCGA pan-cancer dataset)

Cohort5 comprised 10,535 bulk transcriptome samples from 33 cancer types, obtained from the TCGA Pan-Cancer Atlas Hub on UCSC Xena (https://xenabrowser.net/datapages/?cohort=TCGA%20Pan-Cancer%20(PANCAN)&removeHub=https%3A%2F%2Fxena.treehouse.gi.ucsc.edu%3A443). The “batch effects normalized mRNA data” were used for gene expression analysis, and the “curated clinical data” were used for clinical phenotype analysis.

### Pre-processing for single-cell data

Gene-cell matrices were obtained from public databases and filtered based on two criteria: 1) the number of detected genes per cell (excluding cells with <200 or >6,000 genes), and 2) the percentage of mitochondrial UMI counts (excluding cells with >20% mitochondrial UMI counts). UMI normalization was performed by dividing the UMI counts by the total UMI counts in each cell, then multiplying by the universal total UMI count across cells. Finally, UMI counts were transformed into a natural logarithmic scale.

### Raw data processing of single-cell spatial transcriptomics from stereo-seq

GEM files of 4 spatial transcriptomics samples (ST-NR1, ST-NR2, ST-CR1, ST-CR2) containing DNB coordinates and gene UMI counts were collected from previous studies [31]. To obtain expression data at the sub-cellular resolution of 220 nm (one DNB), we merged 50 × 50 DNBs into a single ‘bin’ (25 μm × 25 μm) as the minimum unit for downstream analysis. Bins with fewer than 100 expressed genes or mitochondrial gene counts exceeding 20% were excluded from further analysis.

### Copy Number Variants (CNVs) detection

We implemented an integrative approach combining copy number variant (CNV) detection and expression clustering to identify malignant populations and patterns. The *inferCNV* tool (https://github.com/broadinstitute/inferCNV) was employed to detect somatic alterations of large-scale chromosomal CNVs in individual cells. Reads from immune cells served as the reference for somatic variant determination. The *inferCNV* analysis was conducted using the following parameters: “denoise” function, default hidden Markov model (HMM) settings, and a gene cutoff of 0.1. Cells were subsequently clustered based on their CNV patterns using hierarchical clustering methodology.

### Dimension reduction, clustering, and identification of differentially expressed genes in single-cell data

Dimension reduction was performed using PCA and t-SNE/UMAP implemented in *Seurat* [34] (v5.2.1), based on the transcriptomic data of Cohort 1, Cohort 2 and Cohort 3. We selected 5,000 highly variable genes based on normalized dispersion. The top 30 principal components were used for t-SNE/UMAP projection and clustering analysis, as determined by the elbow plot of standard deviation explained by PCs. Single-cell clustering was conducted using KNN graph construction and the Louvain algorithm with a resolution parameter of 0.5-1.5 (for major cell types, resolution = 0.5; for subtypes of major cell types, resolution = 1.5). Clusters with few differentially expressed genes were merged to prevent over-classification. Differentially expressed genes between clusters were identified using *Seurat*’s *FindAllMarkers* function with the following parameters: pos = TRUE, min.pct = 0.25, logfc.threshold = 0.25, test.use = “wilcox.” Genes with adjusted p < 0.05 were considered differentially expressed.

We used UMAP or t-SNE for different presentation purposes. For visualization of major cell compartments (e.g., epithelial cells, immune cells, and stromal cells), we used UMAP, which provided clearer separation between broad cell populations and improved visualization of global cellular organization. For detailed annotation of subpopulations within specific cell compartments, we used t-SNE, which better highlights local structures and similarities among closely related cell states.

### Identification of transcriptional programs and gene modules using NMF

We employed non-negative matrix factorization (NMF) [35] to identify robust transcriptional programs in all epithelial cells. The expression matrix (genes × cells) was factorized into two matrices (gene × program and program × cell) to infer expression programs. The NMF rank parameter was set to 40 to allow for moderate redundancy. NMF was performed using the R “*snmf/r*” method with “nndsvd” random seed initialization. We sorted gene weights in the obtained transcriptional programs and extracted the top 50 genes with highest weights to construct a 50 × 40 gene-by-program matrix for each individual sample. After merging redundant programs within each sample, we integrated transcriptional programs across all samples and further consolidated redundant programs that shared more than 70% overlapping genes. Finally, a total of 318 robust NMF-derived gene expression programs were identified. We then calculated Jaccard similarity among these programs and performed unsupervised clustering across samples to define program modules. Ultimately, six core meta-programs (MPs) were identified. Gene ontology (GO) analysis was conducted using GO and KEGG databases, and gene set enrichment analysis (GSEA) was performed on hallmark gene sets from MSigDB, ultimately yielding annotated epithelial expression programs and gene modules.

### Calculation of therapeutic Index (Ti)

To quantify the association between changes in cell population abundance and treatment response, we calculated a Therapeutic Index (Ti) [36] for each cell population. Ti was defined as:


$$\begin{array}{c} Ti=sign(slope)\times R^{\text{2}} \end{array}$$ (Equation 1)

where the slope and $R^{\text{2}}$ were obtained from a simple linear regression model, with tumor regression grade (TRG, treated as a numerical variable ranging from 0 to 3) as the dependent variable and the change in resistant cell proportion (ΔRes%, defined as the difference in resistant cell abundance between post-treatment and pre-treatment samples) as the independent variable.

The sign of the slope indicates the direction of the association between cell population dynamics and treatment response, whereas $R^{\text{2}}$ represents the goodness of fit of the linear regression model. A positive Ti value indicates that an increased abundance of the corresponding cell population is associated with poorer treatment response, whereas a negative Ti indicates an association with improved response.

### Gene set enrichment analysis

The GSEA approach was specifically designed to address the high dropout rate in single-cell data and reduce false positives through the following steps: (1) Gene expression was averaged from ten randomly selected cells per cluster; (2) A recovery curve was created, with area under the curve (AUC) computed for the top 3,000 ranked genes; (3) Statistical significance was quantified by comparing target gene sets to 1,000 randomly sampled artificial gene sets of equal size; (4) Gene sets were selected from the Molecular Signatures Database (MSigDB).

### Gene regulatory network analysis based on SCENIC

We applied *pySCENIC* [37] (v0.12.1) to infer transcriptional regulatory networks in tumor cells. Using the single-cell expression matrix as input, transcription factor-target relationships were first derived with *GRNBoost2*. Motif enrichment was then assessed with the cisTarget database to define regulons, and *AUCell* was used to quantify regulon activity per cell. Mean activity scores across tumor cell subpopulations were calculated to identify key transcription factors and their regulatory modules.

### Construction of regulatory networks by igraph

We used the *igraph* package (v2.1.4) in R to construct undirected interaction networks from inferred transcription factor (TF)-target relationships. The network included TFs and targets as nodes, with regulatory relationships represented as edges. Node size was scaled according to the average expression level in the Res_7SK subclones. To optimize visualization, all node sizes were further normalized by a factor of 10. The network layout was generated using the Davidson-Harel algorithm implemented in layout_with_dh function.

### Correlation analysis

Correlation analyses among genes and pathways were performed at either the sample level or the single-cell level. At the sample level, gene or pathway expression was defined as the average expression across all cells within each sample. To ensure data reliability, samples containing fewer than 100 valid targeted cells were excluded to minimize potential biases caused by sparse gene expression data.

At the single-cell level, given the inherent sparsity and zero inflation of single-cell sequencing data, as well as the systematic correlation bias that can arise during normalization, we aggregated every 10 randomly selected cells into one meta-cell. This approach improved data quality and reduced systematic correlation. For correlation analyses, we applied the *stat_cor* function from the *ggpubr* R package (v0.6.1) to calculate Pearson correlation coefficients. Linear regression fitting was performed using the generalized linear model (GLM) approach implemented by the *geom_smooth* function in the *ggplot2* R package (v3.5.2).

Correlation analyses were performed at distinct levels depending on the feature types. Gene-gene and gene-pathway correlations were assessed at the single-cell level to leverage the large number of cells and to capture cell‑level expression relationships. In contrast, pathway-pathway correlations were calculated at the sample level, as single‑cell‑level analysis of such relationships would introduce background positive correlations arising from shared gene expression patterns. Sample‑level aggregation thus provides a more robust evaluation of coordinated changes between gene programs.

### Cell trajectory analysis

To investigate the dynamic changes of tumor cells before and after treatment, we performed trajectory inference using *SpaTrack* [38] (v1.0.2). *SpaTrack* leverages optimal transport to integrate both gene expression and cell positions from scRNA-seq or ST data into transition costs, thereby reconstructing cell differentiation trajectories. In our analysis, tumor cells prior to treatment were defined as the trajectory starting points. During gene preprocessing, we applied filtering thresholds of min_cells > 10 and mean_expr > 0.01. For vector field velocity estimation, the parameters were set to n_neigh_pos = 200 and smooth = 0.8. To identify key genes driving state transitions, we applied further filtering (min_exp_prop = 0.3, hvg_gene = 5000). The top five genes positively and negatively correlated with differentiation trajectories were then selected for analysis and visualization. Statistical significance was defined as P_FDR_ < 0.01.

### Causal testing of the regulatory models

To test hypotheses on the regulatory mechanisms of drug-resistant tumor cells, we performed both Granger causality test [39] and transfer entropy [40] analysis. Cell latent time was estimated with SpaTrack and divided into 10 intervals for Granger causality test and 50 intervals for transfer entropy analysis.

#### Granger causality test.

The Granger causality test evaluates whether past values of one time series can improve the prediction of another time series beyond its own past information. Formally, let $X_{t}$ and $Y_{t}$ be two stationary time series. The test compares the following autoregressive models:


$$\begin{array}{c} Y_{t}=\;\alpha \;+\;\sum_{i=\text{1}}^{p}\beta_{i}Y_{t-i}+\;\epsilon_{t} \end{array}$$ (Equation 2)

$$\begin{array}{c} Y_{t}=\;\alpha \;+\;\sum_{i=\text{1}}^{p}\beta_{i}Y_{t-i}+\;\sum_{j=\text{1}}^{p}\gamma_{j}X_{t-j}+\;u_{t} \end{array}$$ (Equation 3)

where p is the lag order, $\epsilon_{t}$ and $u_{t}$ are error terms. If the variance of $u_{t}$ is significantly smaller than that of $\epsilon_{t}$, i.e., including lagged terms of $X_{t}$ improves the prediction of $Y_{t}$, then $X_{t}$ is said to Granger-cause $Y_{t}$. Conversely, if no significant improvement is observed, we conclude that $X_{t}$ does not Granger-cause $Y_{t}$.

For Granger causality test, we applied the *grangertest* function from the R *lmtest* package to examine causal relationships among 7SK, transcription factors (TFs), and target genes, with the lag order set to 2.

#### Transfer entropy calculation.

Transfer entropy (TE) was used to quantify the directed flow of information between two time series. For two stochastic processes X and Y, the transfer entropy from X to Y is defined as:


$$\begin{array}{c} TE_{\left\{X\;\to \;Y\right\}}=\;\sum p\left(y_{\left\{t+\text{1}\right\}},\;y_{t}^{\left\{(k)\right\}},\;x_{t}^{\left\{(l)\right\}}\right)log_{\text{2}}\frac{p\left(y_{\left\{t+\text{1}\right\}}|y_{t}^{\left\{(k)\right\}},\;x_{t}^{\left\{(l)\right\}}\right)}{p\left(y_{\left\{t+\text{1}\right\}}|y_{t}^{\left\{(k)\right\}}\right)} \end{array}$$ (Equation 4)

Here, $y_{\left\{t+\text{1}\right\}}$ represents the future state of Y; $y_{t}^{\left\{(k)\right\}}$ denotes the past k states of Y; and $x_{t}^{\left\{(l)\right\}}$ denotes the past l states of X. This measure captures how much knowing the past of X reduces the uncertainty in predicting the future of Y, beyond the information already provided by the past of Y. A higher $TE_{\left\{X\;\to \;Y\right\}}$ value indicates a stronger directional influence from X to Y.

For transfer entropy, we used the *transfer_entropy* function from the R package *RTransferEntropy* to estimate both the direction and magnitude of information flow. Pseudotime was split into 50 bins, and bins with fewer than 10 cells were excluded. The lag order was set to 1. Because transfer entropy estimation is sensitive to the number of time points, we repeated the computation 100 times, randomly removing two time points in each run. Finally, we assessed the distribution and statistical significance of the effective transfer entropy.

### Measurement of cellular transcriptional diversity

To quantify the transcriptional diversity of individual cells, we computed the Shannon entropy of each cell’s gene expression profile. The entropy of each single cell is defined as:


$$\begin{array}{c} Entropy\;=-\sum_{i=\text{1}}^{n}p(x_{i}){log}_{\text{2}}p(x_{i}) \end{array}$$ (Equation 5)

where$\;p(x_{i})$ represents the probability of gene expression ${x=x}_{i}$, and n is the number of genes. The higher values indicate a more dispersed and unordered transcriptome, typically associated with greater cellular plasticity or stemness, while lower values reflect a more focused expression program, characteristic of committed and differentiated cell states. The calculation was conducted using *entropy* function of python library *scipy* (v1.10.1).

### Cellular communication analysis

We used *CellChat* [41] (v2.1.2) to analyze cellular communication, with tumor cell subtypes (Res_7SK, Res_other and Sens) as sender cells and tumor microenvironment (TME) cells, including immune cells, endothelial cells and fibroblasts, as receiver cells. Standardized single-cell expression matrices and cell grouping information were used as input. Genes with the lowest 10% expression levels were excluded (trim = 0.1), and only cell groups containing ≥3 cells were retained (min.cells = 3). The analysis employed the default ligand-receptor pair database and signaling pathway annotations provided by *CellChat*. Results were filtered using statistical thresholds (p < 0.001 and communication probability > 0.05). Significant interactions were visualized with heatmaps generated by the R *ComplexHeatmap* package (v2.24.1) using the *ComplexHeatmap* function.

### Cell type identification and deconvolution of spatial transcriptomics data

Cell types in spatial transcriptomics (ST) data were deconvoluted using *SPOTlight* [42] (v1.6.3) with reference to our single-cell data. A preliminary deconvolution of major cell types was carried out using the threshold of 0.3; subsequently, within the identified major cell types, *SPOTlight* was further applied to resolve the composition of subtypes.

### Microenvironmental cell enrichment around Res_7SK and Sens in ST data

We defined cell colocalization as having a Euclidean distance of r < 2 (approximately 50 μm) in ST samples. For cell sets A and B, we counted A cells with any B cell within the cutoff distance. To enable comparative analysis of colocalization between samples, we adjusted values by population size to account for batch differences. Spatial proximity was quantified as the proportion of B cells around A cells within distance r, averaged across all A cells, representing the probability of B cells being within r distance of A cells.

To compare microenvironmental cell enrichment around Res_7SK versus Sens, we calculated the odds ratio (OR) using a 2 × 2 contingency table. The four elements of the table were: (i) the number of target immune cells colocalized with Res_7SK, (ii) the number of other immune cells colocalized with Res_7SK, (iii) the number of target immune cells colocalized with Sens, and (iv) the number of other immune cells colocalized with Sens. We then applied Fisher’s exact test (via the *fisher.test* function) to obtain both the OR and the associated p-value. We examined four ST samples for spatial enrichment of microenvironmental cells. However, only the two NR samples (ST-NR1 and ST-NR2) are shown, because the CR samples contained very few residual tumor cells for reliable spatial co-localization analysis.

### Survival analysis

TCGA gene expression and clinical data were obtained from the UCSC Xena database. Gene scores were calculated by averaging signature expressions, and patients were divided into two groups based on median gene scores for overall survival analysis. Kaplan-Meier curves were generated using *survfit* and *survdiff* functions in R package, with p-values calculated using the log-rank test.

### Screening Res_7SK/Sens tumor subclone signatures

Signatures for resistant and Sens tumor subclones were identified through differential gene expression analysis, comparing each subclone against all others. All identified signatures were ranked according to their fold change (FC) values and systematically compared between resistant and Sens subclones. For each subclone, the top 20 genes exhibiting the highest fold changes were selected as representative signatures.

### Identification of Res_7SK/Sens tumor subclones in validation cohorts

We applied the *AddModuleScore* function from the *Seurat* (v5.2.1) package to identify Res_7SK/Sens tumor subclones in validation cohorts. Specifically, scores of resistant and Sens tumor subclone signatures were calculated as the average expression levels at single-cell resolution, subtracted by the aggregated expression of control feature sets. All analyzed features were binned based on averaged expression, and control features were randomly selected from each bin. Cell populations with statistically significant scores (adjusted p-value < 0.05 and score > 0.05) were identified as target subclones.

### Cell-based functional assays

#### Cell culture and transfection.

Human colorectal cancer cell lines HCT-15 and LS180 were obtained from the American Type Culture Collection (ATCC) and cultured in RPMI-1640 medium (Gibco, USA) supplemented with 10% fetal bovine serum (FBS; Gibco, USA) and 1% penicillin-streptomycin at 37 °C with 5% CO_2_. For functional assays, cells were transfected with either OE-7SK plasmid or empty vector control (overexpression group) or with si-7SK or NC-siRNA (knockdown group) using Lipofectamine 3000 (Thermo Fisher, USA) according to the manufacturer’s protocol. Transfection efficiency was validated by qRT-PCR quantification of 7SK expression.

#### Cell proliferation assay.

To evaluate the effect of 7SK on cell proliferation, MTS assays were performed. After 48h of transfection, cells were seeded into 96-well plates at 3 × 10³ cells/well. Proliferation was measured at 0 h, 24 h, 48 h, and 72 h using the CellTiter 96 Aqueous One Solution assay (Promega, USA). At each time point, 20 µL of reagent was added per well, followed by incubation for 4 h at 37 °C. Absorbance was measured at 490 nm using a Benchmark Plus microplate reader (Bio-Rad, USA). Each condition was tested in three independent experiments, with triplicate wells per experiment.

In parallel, apoptosis and necrosis were assessed by flow cytometry. Transfected cells were stained with Annexin V-FITC/PI (BD Biosciences, USA) according to the manufacturer’s instructions. Total cell death (%) was quantified as the percentage of PI ⁺ /Hoechst⁺ double-positive cells relative to total cells. Data were acquired using a BD FACSCanto II flow cytometer and analyzed with FlowJo software (v10.8, BD Biosciences).

#### Chemoresistance assay.

To assess the role of 7SK in chemotherapy resistance, transfected cells were treated with 5-fluorouracil (5-FU, 10 µM, Sigma-Aldrich, USA) or an equivalent volume of DMSO (vehicle control) for 48 h. After treatment, cells were harvested, washed with PBS, and subjected to PI/Hoechst 33342 double staining (PI: 5 µg/mL; Hoechst: 10 µg/mL; Thermo Fisher, USA) for 30 min at room temperature in the dark. Flow cytometry was performed on a BD FACSCanto II system. Total cell death (%) was quantified as the percentage of PI ⁺ /Hoechst⁺ double-positive cells relative to total cells. Each condition was tested in three independent experiments, with triplicate wells per experiment. Negative (untreated), vehicle (DMSO), and positive (5-FU) control groups were included in all experiments.

## Supporting information

S1 Table Samples and cohorts of this study. (PDF)

S2 Table Sample and clinical information of Cohort1. (PDF)

S1 Fig Cellular Composition of the Tumor Microenvironment in Colorectal Cancer, related to Fig 1. A. Copy-number variation (CNV) landscape of tumor epithelial cells. B. Proportional changes of tumor epithelial subtypes before and after treatment. C. Heterogeneous distribution of expression module activities across tumor epithelial cells, with distinct modules corresponding to different clonal subtypes. D. Box plots illustrate the expression differences of MP2, MP3 and MP5 across all epithelial cell subpopulations. E. Sample origin and t-SNE embedding distribution of different clonal subpopulations. F. Proportional sample source composition of each clonal subpopulation. G. Histogram showing the distribution of tumor cell numbers per sample. The x-axis denotes binned tumor cell counts, and the y-axis shows the frequency of samples within each bin. H. Line plot depicting the variation of Ti values and p-values as a function of tumor cell count filtering thresholds applied to each sample. The x-axis indicates the filtering cutoff. Ti values are shown on the left y-axis, while corresponding p-values are plotted on the right y-axis. Sensitivity analysis across thresholds of 100–1,500 epithelial cells per sample confirmed a stable positive correlation between ΔRes% and TRG (Ti > 0.1, P < 0.05 across all thresholds). We therefore adopted ≥500 cells per sample as the inclusion criterion, excluding three patients (IDs 22, 24, 46), with results remaining highly consistent with the full dataset. I. Scatter plots depicting the association between ΔRes% (percent change in residual tumor burden) and tumor regression grade (TRG) based on all samples.
(PDF)

S2 Fig Functional Characterization of Tumor Epithelium in Colorectal Cancer, related to Fig 2. A. Signature genes of expression modules. Module 3 includes key genes such as 7SK and JUN. B. Functional annotation of expression modules, performed using the top 50 signature genes per module.C. Expression distribution of activated transcription factor (TF) networks in chemotherapy-resistant tumor epithelium. D. Functional annotation of the JUN regulatory network.
(PDF)

S3 Fig Functional Validation of 7SK in Proliferation and Chemoresistance, related to Fig 3. A. In Res_7SK clones, 7SK expression positively correlates with cell cycle and proliferation pathways, but not with other tumor hallmarks. B. Functional assays in CRC cell lines (HCT-15, LS180) confirm that 7SK promotes proliferation and chemoresistance. FACS sorting shows enhanced growth upon 7SK overexpression and increased resistance with 7SK overexpression (upper panel), versus sensitization with 7SK knockdown (lower panel). C. Schematic of the Granger causality test, supporting Model 1 (7SK regulates the JUN network). D. Transfer entropy analysis further supports the regulatory role of 7SK on the JUN network.
(PDF)

S4 Fig Res_7SK Clones Shape an Immunosuppressive Microenvironment, related to Fig 4. A. Subtype composition of major cell types in the CRC tumor microenvironment. B. Heatmap displaying marker genes of cell subsets within the microenvironment. C. Correlation analysis reveals a negative association between Res_7SK signature and T cell activation, while Sens signature shows a positive correlation. D. Res_7SK is spatially colocalized with inactive T cells in another ST sample. E. Spatial colocalization analysis indicates that Res_7SK tumor cells are preferentially co-localized with inactive T cell subsets (naïve T, tissue-resident memory T, and central memory T cells).
(PDF)

S5 Fig Recurrence of Res_7SK and Sens clones across malignancies from Cohort4 (scRNA of 11 cancer types). Recurrence of Res_7SK-like and Sens-like clones across malignancies of multiple cancer types from Cohort4.
(PDF)

S6 Fig Robust identification of the Res_7SK transcriptional state after stringent quality control and across biological sexes. A. t-SNE visualization of single-cell transcriptomic profiles before (Pre) and after (Post) applying stringent quality filtering (>2,000 reads per cell). B. t-SNE visualization of cells classified into Res_7SK, Res_other, Sens, and Normal populations after stringent filtering. Feature plot showing 7SK expression demonstrates specific enrichment within the Res_7SK population. Volcano plot displays differentially expressed genes between Res_7SK and other populations. C. Quantification of transcriptional entropy, mitotic spindle activity, and JUN regulatory network activity among Res_7SK, Res_other, and Sens populations. D. Violin plots showing 7SK expression levels across different cellular states in female and male datasets. 7SK expression was selectively enriched in Res_7SK cells in both sexes. E. t-SNE visualization of female- and male-derived datasets showing classification of Res_7SK, Res_other, and Sens populations. F. Venn diagram showing overlap of Res_7SK marker genes identified independently in female and male datasets (average log2FC > 0.5 and adjusted P value <0.05). A total of 220 genes were shared between sexes, including core Res_7SK markers such as 7SK, ZNF277, SYNE2, JUN, JUNB, HES1, SNORD3A, HEXIM1, and LUC7L3. G. Comparison of mitotic spindle activity, JUN regulatory network activity, and transcriptional entropy among Res_7SK, Res_other, and Sens populations in female and male datasets.
(PDF)
