# Supplementary material for: Non-coding RNA 7SK drives tumor resistance by coupling local oncogenic activation with global transcriptional repression
Source: PLoS Comput Biol. 2026 Sep 8;22(9):e1014749. doi: 10.1371/journal.pcbi.1014749 (PMC13568491; doi:10.1371/journal.pcbi.1014749)
Supplement: Supplementary file 1 [file pcbi.1014749.s001.pdf]

S1 Table. Samples and cohorts of this study.

Data index	Cohorts	Study	Primary site	Cancer type	Technology	Samples	Data_source
1	Cohort1	Qin et al. 2023	colorectal	CRC	DNBelabC4, Stereo-seq	65	CNGBdb: CNP0004138
2	Cohort2	Chen et al. 2024	colorectal	CRC	10x	169	GEO:GSE236581
3	Cohort3	Khaliq et al. 2022	colorectal	CRC	10x	23	GEO:GSE200997
4	Cohort4	Bischoff et al. 2021	lung	LUAD	10x	20	3CA
5	Cohort4	Chan et al. 2021	lung	LUSC	10x	37	3CA
6	Cohort4	Guo et al. 2018	lung	LUAD	SmartSeq2	40	3CA
7	Cohort4	Ireland et al. 2020	lung	LUSC	10x	12	3CA
8	Cohort4	Kim et al. 2020	lung	LUAD	10x	14	3CA
9	Cohort4	Laughney et al. 2020	lung	LUAD	10x	17	3CA
10	Cohort4	Maynard et al. 2020	lung	LUAD	SmartSeq2	50	3CA
11	Cohort4	Qian et al. 2020	lung	LUSC	10x	8	3CA
12	Cohort4	Song et al. 2019	lung	LUAD	10x	6	3CA
13	Cohort4	Xing et al. 2021	lung	LUAD	10x	31	3CA
14	Cohort4	Zilionis et al. 2019	lung	LUAD	inDrop	7	3CA
15	Cohort4	Lu et al. 2020	liver	LIHC	10X	22	GEO:GSE149614
16	Cohort4	Ma et al. 2019	liver	CHOL	10x	8	3CA
17	Cohort4	Sharma et al. 2020	liver	LIHC	10x	58	3CA
18	Cohort4	Sun et al. 2021	liver	LIHC	10x	18	3CA
19	Cohort4	Zhang et al. 2019	liver	LIHC	10x, SmartSeq2	42	3CA
20	Cohort4	Zheng et al. 2017	liver	LIHC	10x	17	3CA
21	Cohort4	Bi et al. 2021	kidney	KIRC	10x	8	3CA
22	Cohort4	Krishna et al. 2021	kidney	KIRC	10x	29	3CA
23	Cohort4	Obradovic et al. 2021	kidney	KIRC	10x	8	3CA
24	Cohort4	Young et al. 2018	kidney	KIRC	10x	66	3CA
25	Cohort4	Zhang et al. 2021	kidney	KIRC	10x	14	3CA
26	Cohort4	Chen et al. 2021	prostate	PRAD	10x	13	3CA
27	Cohort4	Dong et al. 2020	prostate	PRAD	10x	6	3CA
28	Cohort4	He et al. 2021	prostate	PRAD	SmartSeq2	15	3CA
29	Cohort4	Song et al. 2022	prostate	PRAD	Seq-Well S3	15	3CA
30	Cohort4	Chen et al. 2020	head and neck	HNSC	10x	14	3CA
31	Cohort4	Cillo et al. 2020	head and neck	HNSC	10x	48	3CA
32	Cohort4	Kürten et al. 2021	head and neck	HNSC	10x	33	3CA
33	Cohort4	Puram et al. 2017	head and neck	HNSC	SmartSeq2	18	3CA
34	Cohort4	Azizi et al. 2018	breast	BRCA	10x, InDrop	58	3CA
35	Cohort4	Bassez et al. 2021	breast	BRCA	10x	42	3CA
36	Cohort4	Chung et al. 2017	breast	BRCA	SmartSeq2	13	3CA
37	Cohort4	Gao et al. 2021	breast	BRCA	10x	11	3CA
38	Cohort4	Griffiths et al. 2021	breast	BRCA	10x, iCell8	95	3CA
39	Cohort4	Gulati et al. 2020	breast	BRCA	SmartSeq2	15	3CA
40	Cohort4	Jordan et al. 2016	breast	BRCA	unknown	2	3CA
41	Cohort4	Karaayvas et al. 2018	breast	BRCA	SmartSeq2	6	3CA
42	Cohort4	Kim et al. 2018	breast	BRCA	Nanogrid	16	3CA
43	Cohort4	Pal et al. 2021	breast	BRCA	10x	45	3CA
44	Cohort4	Qian et al. 2020	breast	BRCA	10x	7	3CA
45	Cohort4	Savas et al. 2018	breast	BRCA	10x	2	3CA
46	Cohort4	Wu et al. 2021	breast	BRCA	10x	26	3CA
47	Cohort4	Hwang et al. 2022	pancreas	PAAD	10x	43	3CA
48	Cohort4	Lin et al. 2020	pancreas	PAAD	10x	16	3CA

49	Cohort4	Moncada et al. 2020	pancreas	PAAD	inDrop	2	3CA
50	Cohort4	Peng et al. 2019	pancreas	PAAD	10x	16	3CA
51	Cohort4	Raghavan et al. 2021	pancreas	PAAD	Seq-Well	24	3CA
52	Cohort4	Steele et al. 2020	pancreas	PAAD	10x	16	3CA
53	Cohort4	Yue et al. 2020	pancreas	PAAD	10x	15	3CA
54	Cohort4	Geistlinger et al. 2020	ovarian	OV	10x	5	3CA
55	Cohort4	Izar et al. 2020	ovarian	OV	10x, SmartSeq2	14	3CA
56	Cohort4	Nath et al. 2021	ovarian	OV	10x, iCell8	18	3CA
57	Cohort4	Olalekan et al. 2021	ovarian	OV	Drop-seq	6	3CA
58	Cohort4	Olbrecht et al. 2021	ovarian	OV	10x	11	3CA
59	Cohort4	Qian et al. 2020	ovarian	OV	10x	4	3CA
60	Cohort4	Regner et al. 2021	ovarian	OV	10x	11	3CA
61	Cohort4	Shih et al. 2018	ovarian	OV	SmartSeq2	18	3CA
62	Cohort4	Tang et al. 2018	ovarian	OV	10x	4	3CA
63	Cohort4	Zhang et al. 2019	ovarian	OV	10x	2	3CA
64	Cohort4	Zhang et al. 2022	ovarian	OV	10x	22	3CA
65	Cohort4	Biermann et al. 2022	skin	SKCM	10x	32	3CA
66	Cohort4	Andrade et al. 2019	skin	SKCM	10x	12	3CA
67	Cohort4	Arnon et al. 2018	skin	SKCM	SmartSeq2	32	3CA
68	Cohort4	Ji et al. 2020	skin	SKCM	10x	10	3CA
69	Cohort4	Li et al. 2019	skin	SKCM	MARS-seq	27	3CA
70	Cohort4	Mahuron et al. 2020	skin	SKCM	10x	6	3CA
71	Cohort4	Paulson et al. 2020	skin	SKCM	10x	8	3CA
72	Cohort4	Feldman et al. 2018	skin	SKCM	SmartSeq2	48	3CA
73	Cohort4	Tirosh et al. 2016	skin	SKCM	SmartSeq2	19	3CA
74	Cohort4	Yost et al. 2019	skin	SKCM	10x	30	3CA
75	Cohort5	TCGA	pan-cancer	33 cancer types	bulk RNA	10533	UCSC Xena
