# Supplementary material for: Non-coding RNA 7SK drives tumor resistance by coupling local oncogenic activation with global transcriptional repression
Source: PLoS Comput Biol. 2026 Sep 8;22(9):e1014749. doi: 10.1371/journal.pcbi.1014749 (PMC13568491; doi:10.1371/journal.pcbi.1014749)
Supplement: Supplementary file 2 [file pcbi.1014749.s002.pdf]

S2 Table. Sample and clinical information of Cohort1.

Patient ID	Age	Sex	Diagnosis	Molecular subtype	NAC	Tumor regression grade	Tumor response group	Sample before NAC	Sample after NAC
XFZ02	36	M	Rectal adenocarcinoma	MSS/ pMMR	FOLFOX	2	PR	scRNA	scRNA
XFZ03	44	M	Rectal adenocarcinoma	MSS/ pMMR	FOLFOX/ FOLFOXIRI+Bevacizumab	2	PR	scRNA	scRNA
XFZ05	52	M	Rectal adenocarcinoma	MSS/ pMMR	FOLFOX	3	NR	scRNA	scRNA,ST
XFZ07	52	M	Rectal adenocarcinoma	MSS/ pMMR	FOLFOX	0	CR	scRNA	scRNA
XFZ08	65	F	Rectal adenocarcinoma	MSS/ pMMR	FOLFOX	2	PR	scRNA	scRNA
XFZ09	49	M	Rectal adenocarcinoma	MSS/ pMMR	FOLFOX	2	PR	scRNA	scRNA
XFZ17	70	F	Rectal adenocarcinoma	MSS/ pMMR	FOLFOX	2	PR	scRNA	scRNA
XFZ18	63	F	Rectal adenocarcinoma	MSS/ pMMR	FOLFOX	2	PR	NA	scRNA
XFZ19	49	F	Rectal adenocarcinoma	MSS/ pMMR	FOLFOXIRI+Bevacizumab/ FOLFOXIRI	1	CR	scRNA	scRNA,ST
XFZ21	59	F	Rectal adenocarcinoma	MSS/ pMMR	FOLFOX+FOLFOXIRI	NA	NA	scRNA	scRNA
XFZ22	68	F	Rectal adenocarcinoma	MSS/ pMMR	FOLFOX	0	CR	scRNA	scRNA
XFZ23	68	M	Rectal adenocarcinoma	MSS/ pMMR	FOLFOX	3	NR	scRNA	scRNA,ST
XFZ24	41	M	Rectal adenocarcinoma	MSS/ pMMR	FOLFOX	1	CR	scRNA	scRNA
XFZ25	74	M	Rectal adenocarcinoma	MSS/ pMMR	FOLFOX	2	PR	scRNA	scRNA
XFZ26	36	F	Rectal adenocarcinoma	MSS/ pMMR	FOLFOX	2	PR	scRNA	scRNA
XFZ27	64	M	Rectal adenocarcinoma	MSS/ pMMR	FOLFOXIRI	2	PR	scRNA	scRNA
XFZ28	22	M	Rectal adenocarcinoma	MSS/ pMMR	FOLFOXIRI+Bevacizumab	1	CR	scRNA	scRNA,ST
XFZ31	65	M	Rectal adenocarcinoma	MSS/ pMMR	FOLFOX	2	PR	scRNA	scRNA
XFZ32	67	M	Rectal adenocarcinoma	MSS/ pMMR	FOLFOXIRI	2	PR	scRNA	scRNA
XFZ34	73	M	Rectal adenocarcinoma	MSS/ pMMR	XELOX	3	NR	scRNA	scRNA
XFZ35	45	F	Rectal adenocarcinoma	MSS/ pMMR	FOLFOX	2	PR	scRNA	scRNA
XFZ36	52	M	Rectal adenocarcinoma	MSS/ pMMR	FOLFOXIRI	NA	NA	scRNA	scRNA
XFZ38	60	M	Rectal adenocarcinoma	MSS/ pMMR	FOLFOXIRI+Bevacizumab	2	PR	scRNA	scRNA
XFZ39	53	F	Rectal adenocarcinoma	MSS/ pMMR	FOLFOX	2	PR	scRNA	scRNA
XFZ41	52	M	Rectal adenocarcinoma	MSS/ pMMR	FOLFOX	0	CR	NA	scRNA
XFZ44	35	F	Rectal adenocarcinoma	MSS/ pMMR	FOLFOX	2	PR	scRNA	scRNA
XFZ46	63	F	Rectal adenocarcinoma	MSS/ pMMR	FOLFOX	3	NR	scRNA	scRNA
XFZ48	43	M	Rectal adenocarcinoma	MSS/ pMMR	FOLFOXIRI	0	CR	scRNA	scRNA
XFZ49	58	F	Rectal adenocarcinoma	MSS/ pMMR	FOLFOX	3	NR	scRNA	scRNA

NAC, neoadjuvant chemotherapy; ST, single cell spatial transcriptomics; NA, not available.
