# Supplementary material for: Non-coding RNA 7SK drives tumor resistance by coupling local oncogenic activation with global transcriptional repression
Source: PLoS Comput Biol. 2026 Sep 8;22(9):e1014749. doi: 10.1371/journal.pcbi.1014749 (PMC13568491; doi:10.1371/journal.pcbi.1014749)
Supplement: Supplementary file 3 [file pcbi.1014749.s003.pdf]

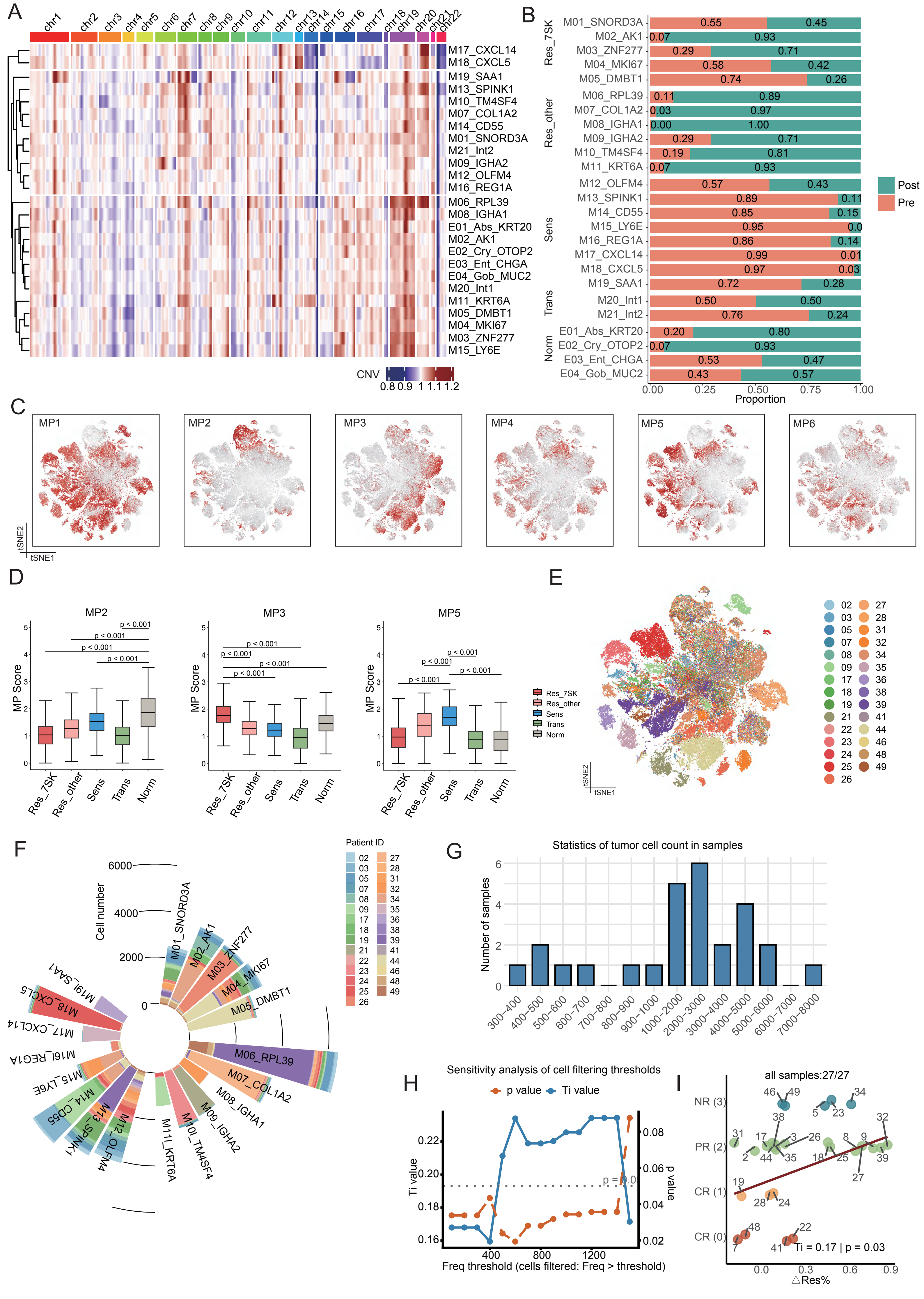
A
chr1 chr2 chr3 chr4 chr5 chr6 chr7 chr8 chr9 chr10 chr11 chr12 chr13 chr14 chr15 chr16 chr17 chr18 chr19 chr20 chr21 chr22
M17_CXCL14
M18_CXCL5
M19_SAA1
M13_SPINK1
M10_TM4SF4
M07_COL1A2
M14_CD55
M01_SNORD3A
M21_Int2
M09_IGHA2
M12_OLFM4
M16_REG1A
M06_RPL39
M08_IGHA1
E01_Abs_KRT20
M02_AK1
E02_Cry_OTOP2
E03_Ent_CHGA
E04_Gob_MUC2
M20_Int1
M11_KRT6A
M05_DMBT1
M04_MKI67
M03_ZNF277
M15_LY6E
CNV
0.8 0.9 1 1.1 1.2
B
Res_7SK
M01_SNORD3A 0.55 0.45
M02_AK1 0.07 0.93
M03_ZNF277 0.29 0.71
M04_MKI67 0.58 0.42
M05_DMBT1 0.74 0.26
Res_other
M06_RPL39 0.11 0.89
M07_COL1A2 0.03 0.97
M08_IGHA1 0.00 1.00
M09_IGHA2 0.29 0.71
M10_TM4SF4 0.19 0.81
M11_KRT6A 0.07 0.93
Sens
M12_OLFM4 0.57 0.43
M13_SPINK1 0.89 0.11
M14_CD55 0.85 0.15
M15_LY6E 0.95 0.0
M16_REG1A 0.86 0.14
M17_CXCL14 0.99 0.01
M18_CXCL5 0.97 0.03
M19_SAA1 0.72 0.28
Trans
M20_Int1 0.50 0.50
M21_Int2 0.76 0.24
Norm
E01_Abs_KRT20 0.20 0.80
E02_Cry_OTOP2 0.07 0.93
E03_Ent_CHGA 0.53 0.47
E04_Gob_MUC2 0.43 0.57
0.00 0.25 0.50 0.75 1.00
Proportion
Post
Pre
C
MP1 MP2 MP3 MP4 MP5 MP6
tSNE2
tSNE1
D
MP2 MP3 MP5
MP Score
p < 0.001
Res_7SK Res_other Sens Trans Norm
E
02 03 05 07 08 09 17 18 19 21 22 23 24 25 26
27 28 31 32 34 35 36 38 39 41 44 46 48 49
tSNE2
tSNE1
F
Cell number
6000 4000 2000 0
M01_SNORD3A M02_AK1 M03_ZNF277 M04_MKI67 M05_DMBT1 M06_RPL39 M07_COL1A2 M08_IGHA1 M09_IGHA2 M10_TM4SF4 M11_KRT6A M12_OLFM4 M13_SPINK1 M14_CD55 M15_LY6E M16_REG1A M17_CXCL14 M18_CXCL5 M19_SAA1
Patient ID
02 03 05 07 08 09 17 18 19 21 22 23 24 25 26
27 28 31 32 34 35 36 38 39 41 44 46 48 49
G
Statistics of tumor cell count in samples
Number of samples
0 2 4 6
300-400 400-500 500-600 600-700 700-800 800-900 900-1000 1000-2000 2000-3000 3000-4000 4000-5000 5000-6000 6000-7000 7000-8000
H
Sensitivity analysis of cell filtering thresholds
p value Ti value
Ti value
p value
0.16 0.18 0.20 0.22
0.02 0.04 0.06 0.08
p = 0.0
400 800 1200
Freq threshold (cells filtered: Freq > threshold)
I
all samples:27/27
NR (3)
PR (2)
CR (1)
CR (0)
46 49 34 5 23 38 31 17 3 26 2 44 35 18 8 9 25 39 27 19 28 24 48 22 7 41
Ti = 0.17 | p = 0.03
0.0 0.3 0.6 0.9
△Res%
