# Supplementary material for: Non-coding RNA 7SK drives tumor resistance by coupling local oncogenic activation with global transcriptional repression
Source: PLoS Comput Biol. 2026 Sep 8;22(9):e1014749. doi: 10.1371/journal.pcbi.1014749 (PMC13568491; doi:10.1371/journal.pcbi.1014749)
Supplement: Supplementary file 4 [file pcbi.1014749.s004.pdf]

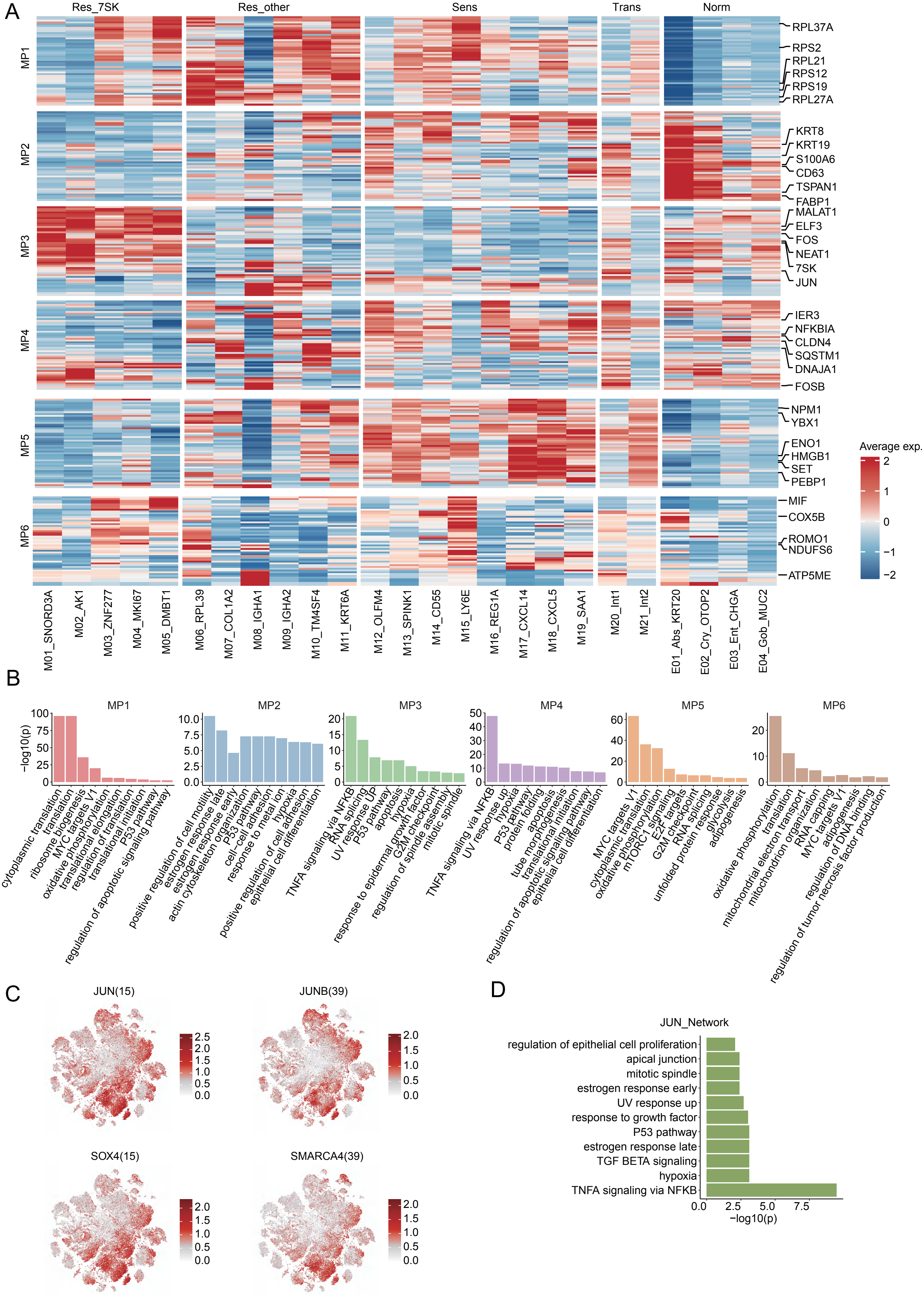
A
Res_7SK
Res_other
Sens
Trans
Norm
MP1
MP2
MP3
MP4
MP5
MP6
RPL37A
RPS2
RPL21
RPS12
RPS19
RPL27A
KRT8
KRT19
S100A6
CD63
TSPAN1
FABP1
MALAT1
ELF3
FOS
NEAT1
7SK
JUN
IER3
NFKBIA
CLDN4
SQSTM1
DNAJA1
FOSB
NPM1
YBX1
ENO1
HMGB1
SET
PEBP1
MIF
COX5B
ROMO1
NDUFS6
ATP5ME
Average exp.
2
1
0
−1
−2
M01_SNORD3A
M02_AK1
M03_ZNF277
M04_MKI67
M05_DMBT1
M06_RPL39
M07_COL1A2
M08_IGHA1
M09_IGHA2
M10_TM4SF4
M11_KRT6A
M12_OLFM4
M13_SPINK1
M14_CD55
M15_LY6E
M16_REG1A
M17_CXCL14
M18_CXCL5
M19_SAA1
M20_Int1
M21_Int2
E01_Abs_KRT20
E02_Cry_OTOP2
E03_Ent_CHGA
E04_Gob_MUC2
B
MP1
−log10(p)
cytoplasmic translation
translation
ribosome biogenesis
MYC targets V1
oxidative phosphorylation
translational elongation
regulation of translation
translational initiation
P53 pathway
regulation of apoptotic signaling pathway
MP2
positive regulation of cell motility
estrogen response late
estrogen response early
actin cytoskeleton organization
P53 pathway
cell−cell adhesion
response to metal ion
hypoxia
positive regulation of cell adhesion
epithelial cell differentiation
MP3
TNFA signaling via NFKB
RNA splicing
UV response UP
P53 pathway
apoptosis
hypoxia
response to epidermal growth factor
G2M checkpoint
regulation of spindle assembly
mitotic spindle
MP4
TNFA signaling via NFKB
UV response up
hypoxia
P53 pathway
protein folding
apoptosis
tube morphogenesis
translational initiation
regulation of apoptotic signaling pathway
epithelial cell differentiation
MP5
MYC targets V1
cytoplasmic translation
oxidative phosphorylation
mTORC1 signaling
E2F targets
G2M checkpoint
RNA splicing
unfolded protein response
glycolysis
adipogenesis
MP6
oxidative phosphorylation
translation
mitochondrial electron transport
mitochondrion organization
RNA capping
MYC targets V1
adipogenesis
regulation of DNA binding
regulation of tumor necrosis factor production
C
JUN(15)
JUNB(39)
SOX4(15)
SMARCA4(39)
D
JUN_Network
regulation of epithelial cell proliferation
apical junction
mitotic spindle
estrogen response early
UV response up
response to growth factor
P53 pathway
estrogen response late
TGF BETA signaling
hypoxia
TNFA signaling via NFKB
0.0
2.5
5.0
7.5
−log10(p)
