# Supplementary material for: Non-coding RNA 7SK drives tumor resistance by coupling local oncogenic activation with global transcriptional repression
Source: PLoS Comput Biol. 2026 Sep 8;22(9):e1014749. doi: 10.1371/journal.pcbi.1014749 (PMC13568491; doi:10.1371/journal.pcbi.1014749)

A

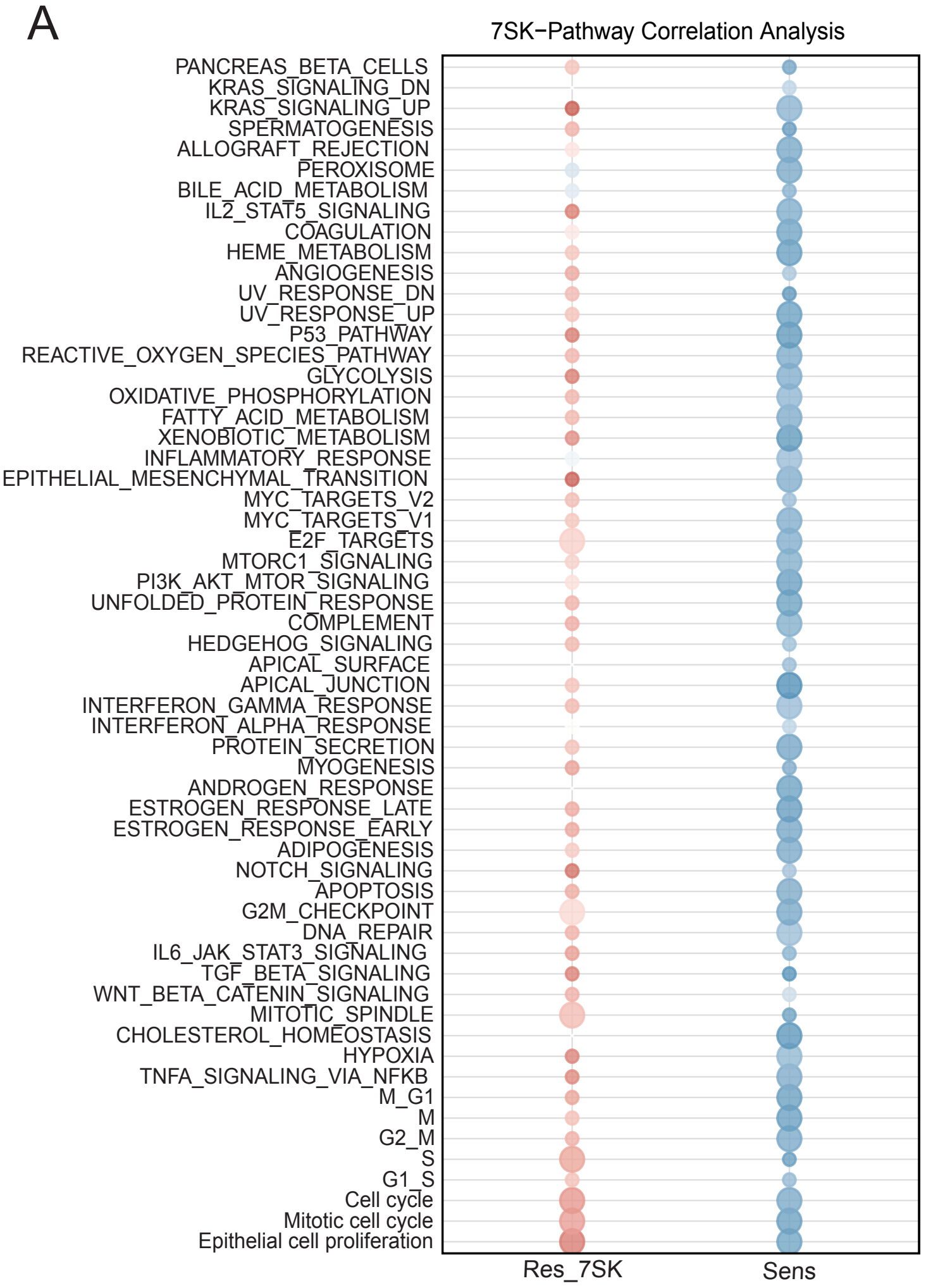

B

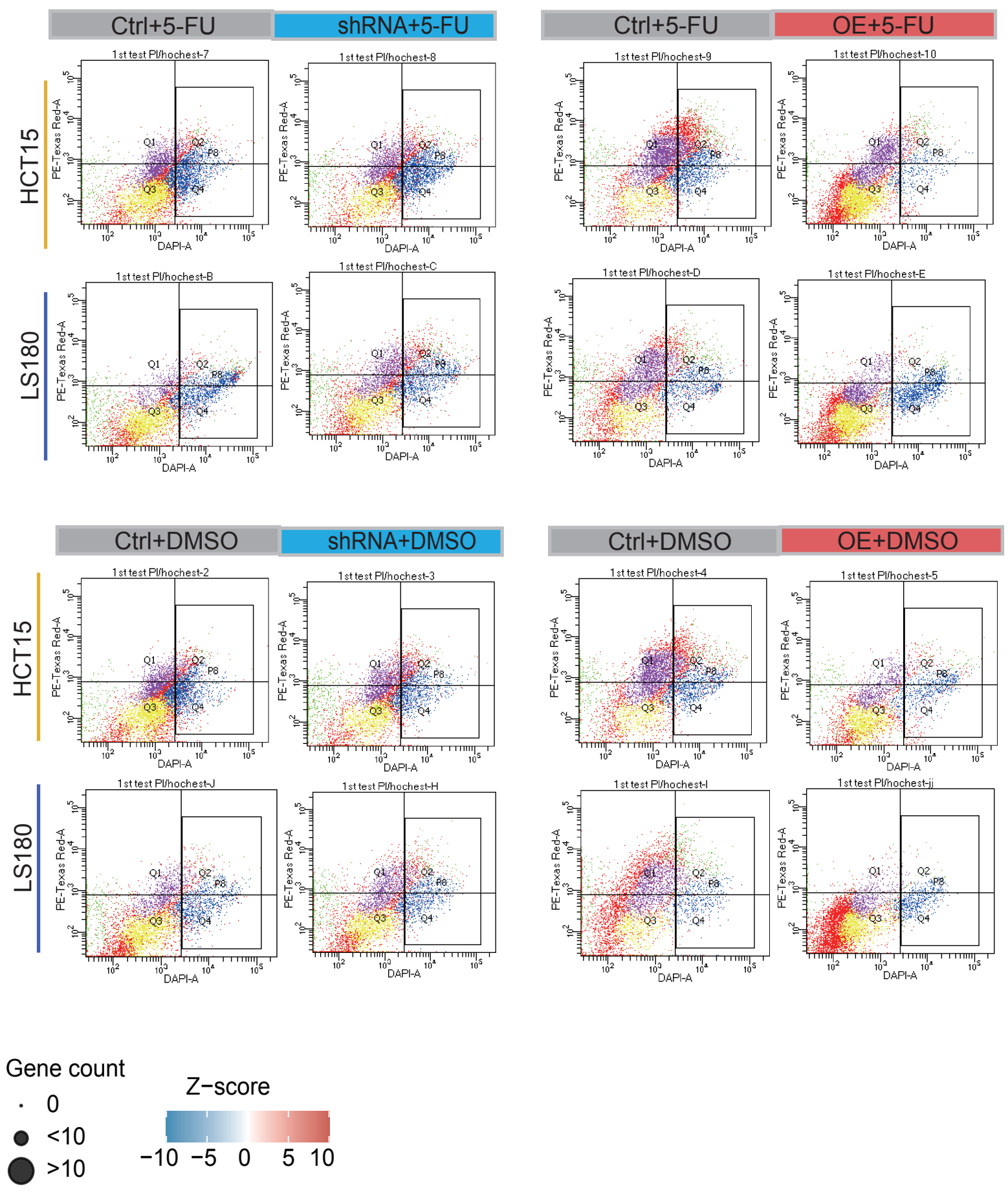

C

gene	Candidate causal gene		Lag order	F value	P value	Significance	Conclusion
JUN	7SK	10	2	6.15	0.087	Marginal	7SK → JUN (marginally significant)
JUN	target	10	2	1.13	0.431	No	target does not predict JUN
7SK	JUN	10	2	2.22	0.256	No	JUN does not predict 7SK
7SK	target	10	2	3.36	0.171	No	target does not predict 7SK
target	JUN	10	2	15.87	0.026	Yes	JUN → target
target	7SK	10	2				

D

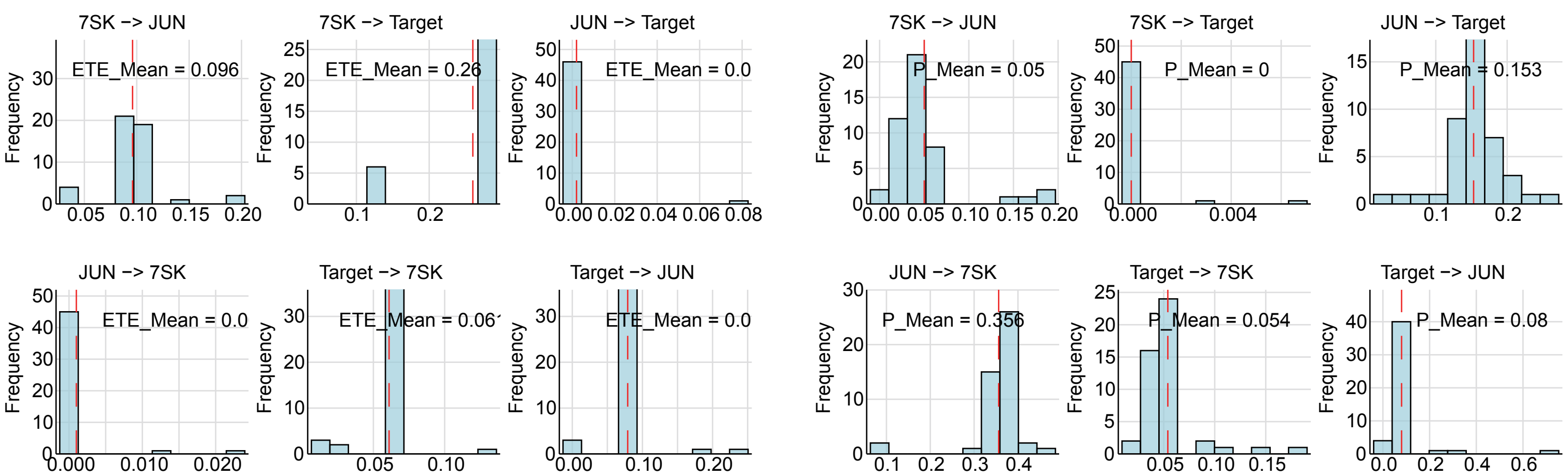

Supplement: Supplementary file 5 [file pcbi.1014749.s005.pdf]
