# Supplementary material for: Non-coding RNA 7SK drives tumor resistance by coupling local oncogenic activation with global transcriptional repression
Source: PLoS Comput Biol. 2026 Sep 8;22(9):e1014749. doi: 10.1371/journal.pcbi.1014749 (PMC13568491; doi:10.1371/journal.pcbi.1014749)
Supplement: Supplementary file 6 [file pcbi.1014749.s006.pdf]

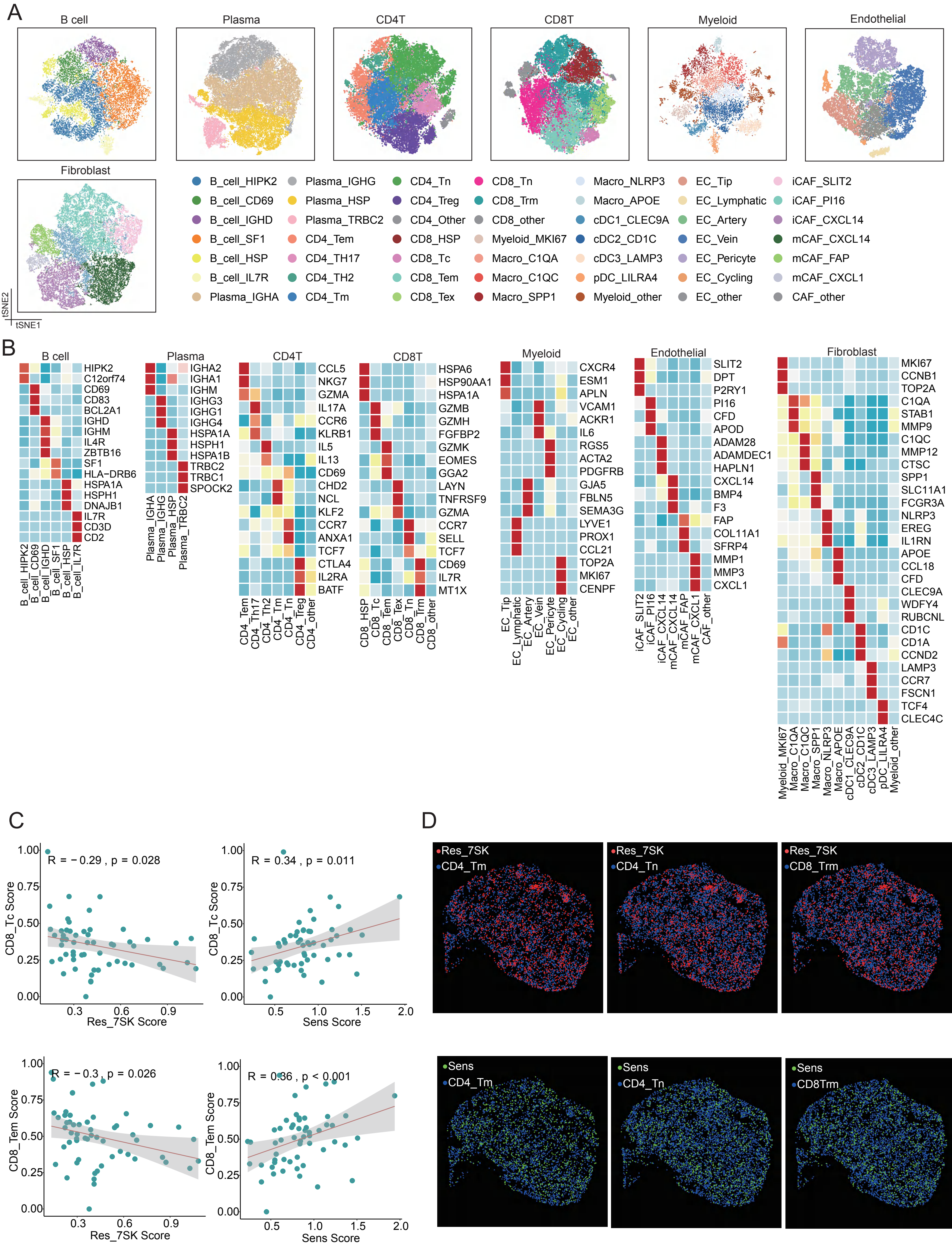
A
B cell
Plasma
CD4T
CD8T
Myeloid
Endothelial
Fibroblast
tSNE2
tSNE1
B_cell_HIPK2
B_cell_CD69
B_cell_IGHD
B_cell_SF1
B_cell_HSP
B_cell_IL7R
Plasma_IGHA
Plasma_IGHG
Plasma_HSP
Plasma_TRBC2
CD4_Tem
CD4_TH17
CD4_TH2
CD4_Tm
CD4_Tn
CD4_Treg
CD4_Other
CD8_HSP
CD8_Tc
CD8_Tem
CD8_Tex
CD8_Tn
CD8_Trm
CD8_other
Myeloid_MKI67
Macro_C1QA
Macro_C1QC
Macro_SPP1
Macro_NLRP3
Macro_APOE
cDC1_CLEC9A
cDC2_CD1C
cDC3_LAMP3
pDC_LILRA4
Myeloid_other
EC_Tip
EC_Lymphatic
EC_Artery
EC_Vein
EC_Pericyte
EC_Cycling
EC_other
iCAF_SLIT2
iCAF_PI16
iCAF_CXCL14
mCAF_CXCL14
mCAF_FAP
mCAF_CXCL1
CAF_other
B
B cell
HIPK2
C12orf74
CD69
CD83
BCL2A1
IGHD
IGHM
IL4R
ZBTB16
SF1
HLA−DRB6
HSPA1A
HSPH1
DNAJB1
IL7R
CD3D
CD2
Plasma
IGHA2
IGHA1
IGHM
IGHG3
IGHG1
IGHG4
HSPA1A
HSPH1
HSPA1B
TRBC2
TRBC1
SPOCK2
CD4T
CCL5
NKG7
GZMA
IL17A
CCR6
KLRB1
IL5
IL13
CD69
CHD2
NCL
KLF2
CCR7
ANXA1
TCF7
CTLA4
IL2RA
BATF
CD8T
HSPA6
HSP90AA1
HSPA1A
GZMB
GZMH
FGFBP2
GZMK
EOMES
GGA2
LAYN
TNFRSF9
GZMA
CCR7
SELL
TCF7
CD69
IL7R
MT1X
Myeloid
CXCR4
ESM1
APLN
VCAM1
ACKR1
IL6
RGS5
ACTA2
PDGFRB
GJA5
FBLN5
SEMA3G
LYVE1
PROX1
CCL21
TOP2A
MKI67
CENPF
Endothelial
SLIT2
DPT
P2RY1
PI16
CFD
APOD
ADAM28
ADAMDEC1
HAPLN1
CXCL14
BMP4
F3
FAP
COL11A1
SFRP4
MMP1
MMP3
CXCL1
Fibroblast
MKI67
CCNB1
TOP2A
C1QA
STAB1
MMP9
C1QC
MMP12
CTSC
SPP1
SLC11A1
FCGR3A
NLRP3
EREG
IL1RN
APOE
CCL18
CFD
CLEC9A
WDFY4
RUBCNL
CD1C
CD1A
CCND2
LAMP3
CCR7
FSCN1
TCF4
CLEC4C
C
R = −0.29 , p = 0.028
CD8_Tc Score
Res_7SK Score
R = 0.34 , p = 0.011
CD8_Tc Score
Sens Score
R = −0.3 , p = 0.026
CD8_Tem Score
Res_7SK Score
R = 0.36 , p < 0.001
CD8_Tem Score
Sens Score
D
Res_7SK
CD4_Tm
Res_7SK
CD4_Tn
Res_7SK
CD8_Trm
Sens
CD4_Tm
Sens
CD4_Tn
Sens
CD8Trm
