# Supplementary material for: Non-coding RNA 7SK drives tumor resistance by coupling local oncogenic activation with global transcriptional repression
Source: PLoS Comput Biol. 2026 Sep 8;22(9):e1014749. doi: 10.1371/journal.pcbi.1014749 (PMC13568491; doi:10.1371/journal.pcbi.1014749)
Supplement: Supplementary file 7 [file pcbi.1014749.s007.pdf]

A

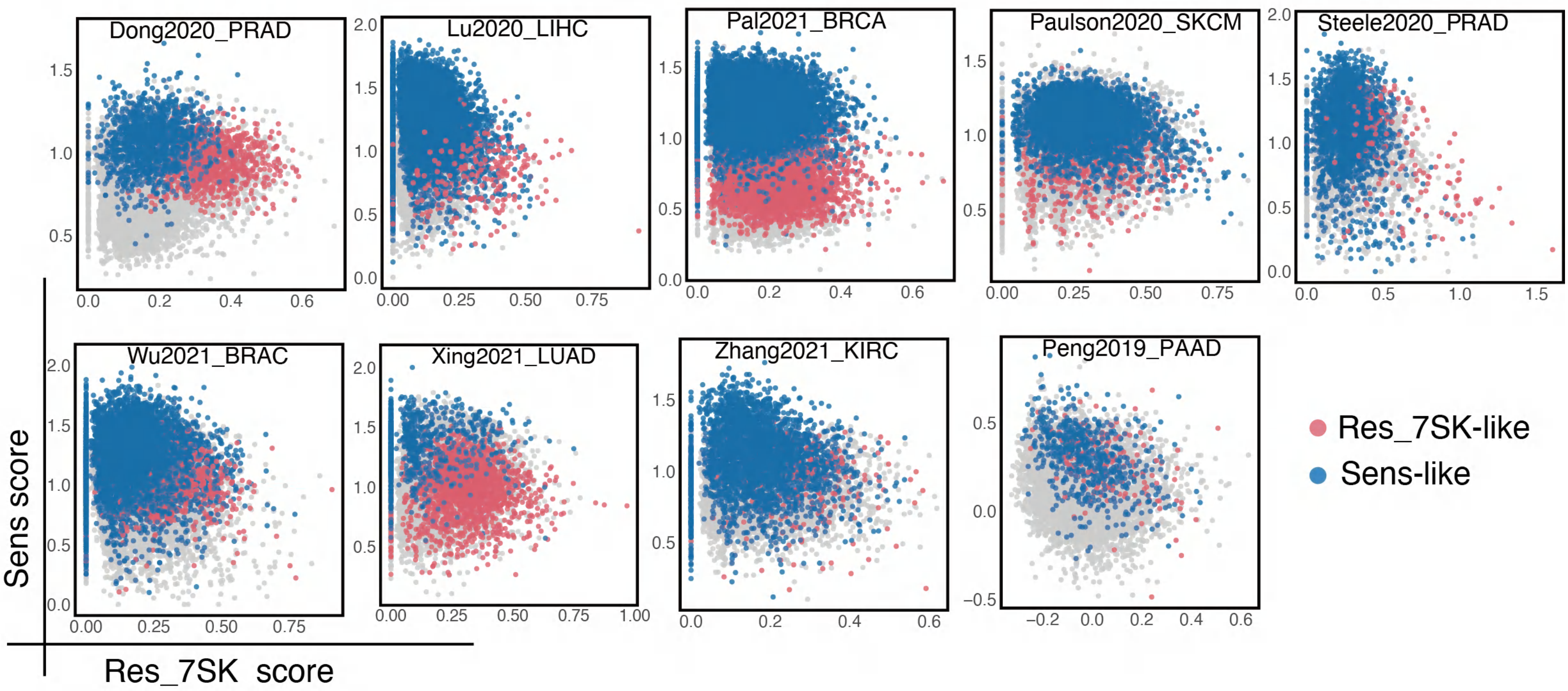
Dong2020_PRAD
Lu2020_LIHC
Pal2021_BRCA
Paulson2020_SKCM
Steele2020_PRAD
Wu2021_BRAC
Xing2021_LUAD
Zhang2021_KIRC
Peng2019_PAAD
Res_7SK-like
Sens-like
Sens score
Res_7SK score
