# Supplementary material for: Non-coding RNA 7SK drives tumor resistance by coupling local oncogenic activation with global transcriptional repression
Source: PLoS Comput Biol. 2026 Sep 8;22(9):e1014749. doi: 10.1371/journal.pcbi.1014749 (PMC13568491; doi:10.1371/journal.pcbi.1014749)
Supplement: Supplementary file 8 [file pcbi.1014749.s008.pdf]

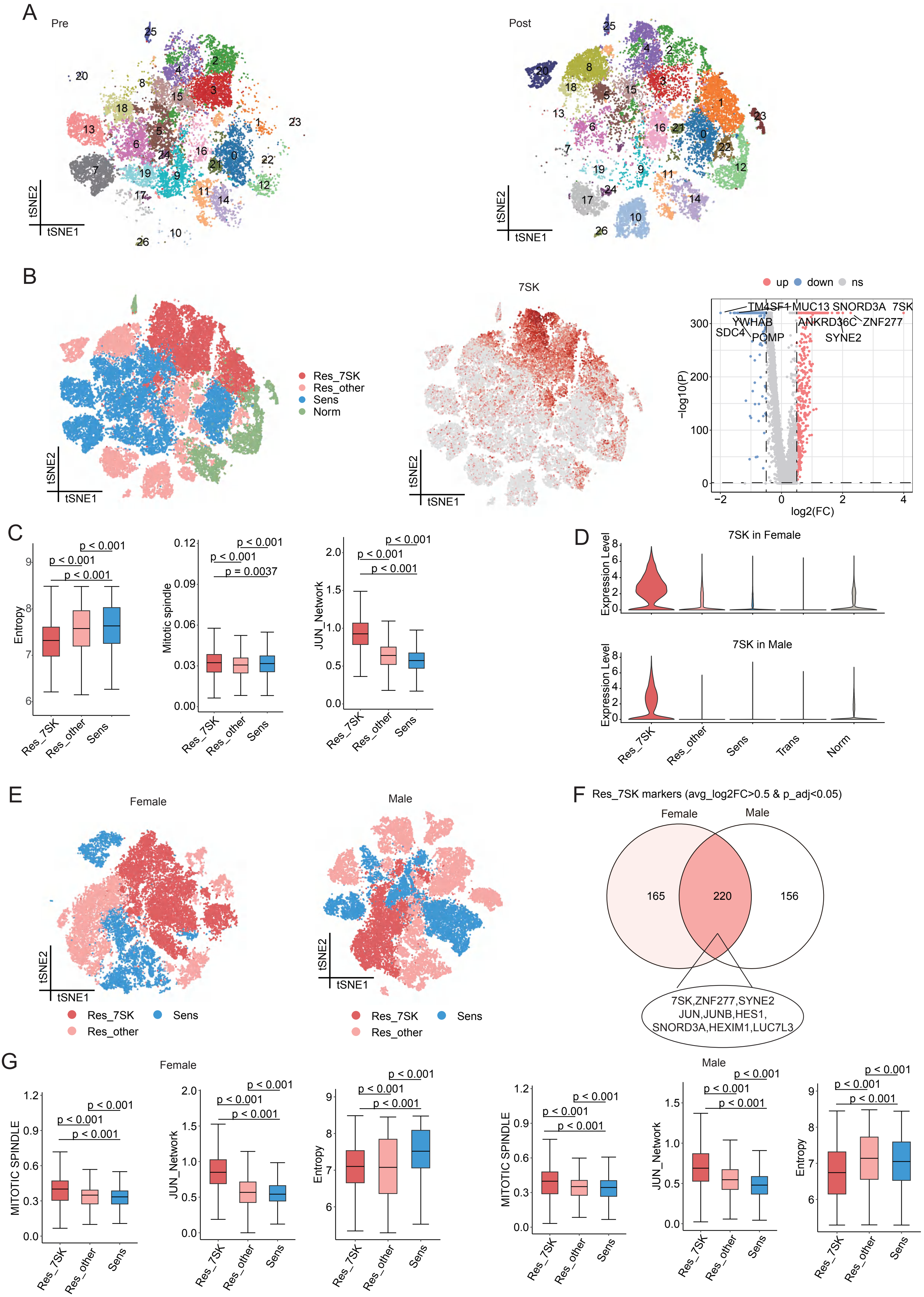

A
Pre
Post
tSNE1
tSNE2
B
Res_7SK
Res_other
Sens
Norm
7SK
up
down
ns
TM4SF1
MUC13
SNORD3A
7SK
YWHAB
ANKRD36C
ZNF277
SDC4
POMP
SYNE2
-log10(P)
log2(FC)
C
Entropy
Mitotic spindle
JUN_Network
p < 0.001
p = 0.0037
D
7SK in Female
7SK in Male
Expression Level
Res_7SK
Res_other
Sens
Trans
Norm
E
Female
Male
F
Res_7SK markers (avg_log2FC>0.5 & p_adj<0.05)
Female
Male
165
220
156
7SK,ZNF277,SYNE2
JUN,JUNB,HES1,
SNORD3A,HEXIM1,LUC7L3
G
Female
Male
MITOTIC SPINDLE
JUN_Network
Entropy
